# Pectoral myology of limb-reduced worm lizards (Squamata, Amphisbaenia) suggests decoupling of the musculoskeletal system during the evolution of body elongation

**DOI:** 10.1186/s12862-018-1303-1

**Published:** 2019-01-10

**Authors:** Natascha Westphal, Kristin Mahlow, Jason James Head, Johannes Müller

**Affiliations:** 1 Museum für Naturkunde Berlin, Leibniz-Institut für Evolutions- und Biodiversitätsforschung, Invalidenstr. 43, 10115 Berlin, Germany; 2 Department of Zoology and University Museum of Zoology, University of Cambridge, Downing St, Cambridge, CB2 3EJ UK

**Keywords:** diceCT, Amphisbaenia, Musculoskeletal system, Pectoral myology, Body elongation, Skeletal reductions

## Abstract

**Background:**

The evolution of elongated body forms in tetrapods has a strong influence on the musculoskeletal system, including the reduction of pelvic and pectoral girdles, as well as the limbs. However, despite extensive research in this area it still remains unknown how muscles within and around bony girdles are affected by these reductions. Here we investigate this issue using fossorial amphisbaenian reptiles, or worm lizards, as a model system, which show substantial variation in the degree of reductions of girdles and limbs. Using iodine-based contrast-enhanced computed tomography (diceCT), we analyze the composition of the shoulder muscles of the main clades of Amphisbaenia and their outgroups relative to the pectoral skeleton.

**Results:**

All investigated amphisbaenian taxa retain the full set of 17 shoulder muscles, independent of the degree of limb and girdle reductions, whereas in some cases muscles are fused to complexes or changed in morphology relative to the ancestral condition. *Bipes* is the only taxon that retains forelimbs and an almost complete pectoral girdle. All other amphisbaenian families show more variation concerning the completeness of the pectoral girdle having reduced or absent girdle elements. *Rhineura,* which undergoes the most severe bone reductions, differs from all other taxa in possessing elongated muscle strands instead of discrete shoulder muscles. In all investigated amphisbaenians, the shoulder muscle agglomerate is shortened and shifted anteriorly relative to the ancestral position as seen in the outgroups*.*

**Conclusions:**

Our results show that pectoral muscle anatomy does not necessarily correspond to the loss or reduction of bones, indicating a decoupling of the musculoskeletal system. Muscle attachment sites change from bones to non-skeletal areas, such as surrounding muscles, skin or connective tissue, whereas muscle origins themselves remain in the same region where the pectoral bones were ancestrally located. Our findings indicate a high degree of developmental autonomy within the musculoskeletal system, we predict that the observed evolutionary rearrangements of amphisbaenian shoulder muscles were driven by functional demands rather than by developmental constraints. Nevertheless, worm lizards display a spatial offset of both pectoral bones and muscles relative to the ancestral position, indicating severe developmental modifications of the amphisbaenian body axis.

**Electronic supplementary material:**

The online version of this article (10.1186/s12862-018-1303-1) contains supplementary material, which is available to authorized users.

## Background

In tetrapods, the evolution of elongated body forms is tied to substantial modifications of the musculoskeletal system and has shown to be strongly correlated with the reduction of limbs [1–7]. Limb reduction runs proximo-distally from the girdles and not only includes the loss of digits and extremities, but may also lead to the complete loss of the bony pectoral and pelvic girdles [8]. Whereas body elongation has been recorded multiple times within fossil and living amphibians and various clades of amniotes and also actinopterygian fishes [9, 10], it is an especially common phenomenon in squamate reptiles, the clade comprising lizards and snakes [11–14]. Many body-elongated taxa within Squamata not only lost their extremities but also most, or even all, of their pelvic and pectoral elements [9, 11], the most famous example being snakes. Whereas patterns of bone reduction have been frequently studied in various clades of lizards [3, 13–19], it still remains poorly understood as to what extent also muscle anatomy is affected by these processes. Previous studies on lizard digit morphology suggest that muscle anatomy does not necessarily correspond to the loss or reduction of bones [6]; a finding that is in concert with developmental investigations on mouse limbs and studies on the normal phenotype of non-pentadactyl animals, and of human birth defects, suggesting that skeletal and connective tissue patterning are decoupled [1, 6, 20–23]. However, it still remains unknown how the musculoskeletal system is integrated along the main body axis, and there is practically no information on muscle anatomy and topology within and around reduced bony girdles. Given that such a fundamental evolutionary transformation as body elongation requires more anatomical changes than the mere reduction of extremities, this lack of knowledge is unfortunate.

In the present study we explore this issue using worm lizards, or Amphisbaenia, as a model system. Amphisbaenians are squamate reptiles highly specialized for a burrowing (fossorial) lifestyle. Next to their snake-like appearance, amphisbaenians are anatomically characterized by a strongly reinforced skull that is used as a digging tool, the loss of the middle ear, and the reduction of the eyes [11, 24–29]. Only a single genus, *Bipes*, possesses forelimbs [8, 30, 31]. In addition to limb reduction, the pectoral and pelvic girdles are reduced within Amphisbaenia. Substantial variation in the degree of reduction occurs across the major clades. There are different levels of reduction in the pectoral girdle, with *Bipes* retaining large parts of the bony shoulder girdle and Blanidae still having ossified clavicles and scapulocoracoids, whereas Trogonophidae only possess bony scapulocoracoids and poorly ossified remnants of the sternum; Amphisbaenidae show either no or only very small bony scapulocoracoids and Rhineuridae do not retain any element of the pectoral girdle (see Table 1) [8, 30, 32]. The presence of variable girdle reduction within Amphisbaenia makes this clade an ideal study system for potentially corresponding changes in bone and muscle structure, as all amphisbaenian families show an elongated and limb-reduced morphology [8]. Previously there have been only a limited amount of studies addressing the presence and morphology of pectoral muscles in Amphisbaenia [33, 34], suggesting that at least a few muscles may be retained in some taxa. More detailed studies, however, have been lacking, which is likely due to the often small size of the animals making careful anatomical dissections difficult, and the lack of a resolved phylogenetic bracket that only improved in recent years [32, 35, 36].

**Table 1:** Presence and condition of different pectoral girdle elements across amphisbaenian families

	Suprascapulae	Clavicles	Sternum	Scapulocoracoids
Bipedidae	calcified	bony	cartilaginous	bony
Blanidae	–	ossified	cartilaginous	ossified
Trogonophidae	–	–	cartilaginous	bony
Amphisbaenidae	–	–	–	bony
Rhineuridae	–	–	–	–

For our study we used diffusible iodine-based contrast-enhanced computed tomography (diceCT), a recently developed technique for the 3D visualization of metazoan soft tissues [37–40]. As we will show, this approach allowed us to comprehensively analyze the pectoral muscle anatomy of Amphisbaenia across all major clades and within a modern phylogenetic context based on the phylogenies by Vidal et al. (2008) [35], Hipsley & Müller (2014) [41], and Pyron et al. (2013) [36]. Lacertidae have been found to be the closest relatives of amphisbaenians [42–44], which is why we used a lacertid lizard, i.e. *Meroles cuneirostris,* as outgroup taxon for comparison.

## Material & Methods

We used a personally developed protocol for diceCT visualization (iodine-potassium iodine solution from stock solution diluted with H_2_O dest; 0,149 g/l in 100 ml H_2_O = ~ 0,15 w/v I in H_2_O), and applied this method to individuals belonging to five amphisbaenian species, *Rhineura floridana, Bipes biporus, Blanus strauchi, Trogonophis wiegmanni* and *Cynisca leucura*, which are representatives of the five major clades of Amphisbaenia, respectively (Rhineuridae: *Rhineura*, Bipedidae: *Bipes*, Blanidae: *Blanus*, Trogonophidae: *Trogonophis*, Amphisbaenidae: *Cynisca*) [45] (see Additional file 1: Table S1). Because lacertid lizards were recently identified as the sister taxon of Amphisbaenia [42–44], we used representatives from this clade (*Meroles cuneirostris*, see Additional file 2: Text S2, Additional file 3: Figure S3 and Additional file 4: Figure S4) as outgroup and reference for pectoral muscle morphology, together with previously published information from other squamates [46]. All specimens were stored in 75% ethanol before staining. They were rehydrated in two-day steps before staining. During staining, the solution was changed every three days during the first three weeks and afterwards when the solution lost colour and a change was necessary. Staining times depend on the size and permeability of the respective specimen, which took 17 days for *C. leucura*, 33 days for *B. biporus*, 72 days for *T. wiegmanni*, 79 days for *R. floridana*, and 86 days for *M. cuneirostris*. After completed staining, specimens were scanned using a Phoenix X-ray Nanotom computed tomography machine at MfN using 90 kV, 110 MA and 1000 ms for *R. floridana* and 40 kV, 250 μA und 1250 ms for all other specimens, with a resolution between 4 and 8 μm. The resulting 3D model was visualized and analysed using the software Volume Graphics 2.0. Muscle terminology follows Jenkins and Goslow (1983) [46]. The identification of the muscles of the shoulder girdle with respect to their adhesion points on bones was not always possible due to the reductions and loss of many ossified shoulder girdle elements in amphisbaenians. Therefore, muscle identification was primarily made on the basis of the topological relationships of the different muscle strands relative to each other.

We used the software Mesquite (version 3.10) [47] to optimize observed muscle characters onto amphisbaenian phylogenetic relationships. We used parsimony to perform the analyses and loaded 33 morphological characters into a matrix and mapped them on the two most conflicting phylogeny hypotheses by Vidal et al. (2008) [35] and Hipsley & Müller (2014) [41] as well as Pyron et al. (2013) [36].

## Results

### Amphisbaenian shoulder muscle anatomy

#### Non-rhineurid Amphisbaenia

##### Bipedidae (*Bipes biporus*)

**Bones of the shoulder girdle**

**Fig. 1:**
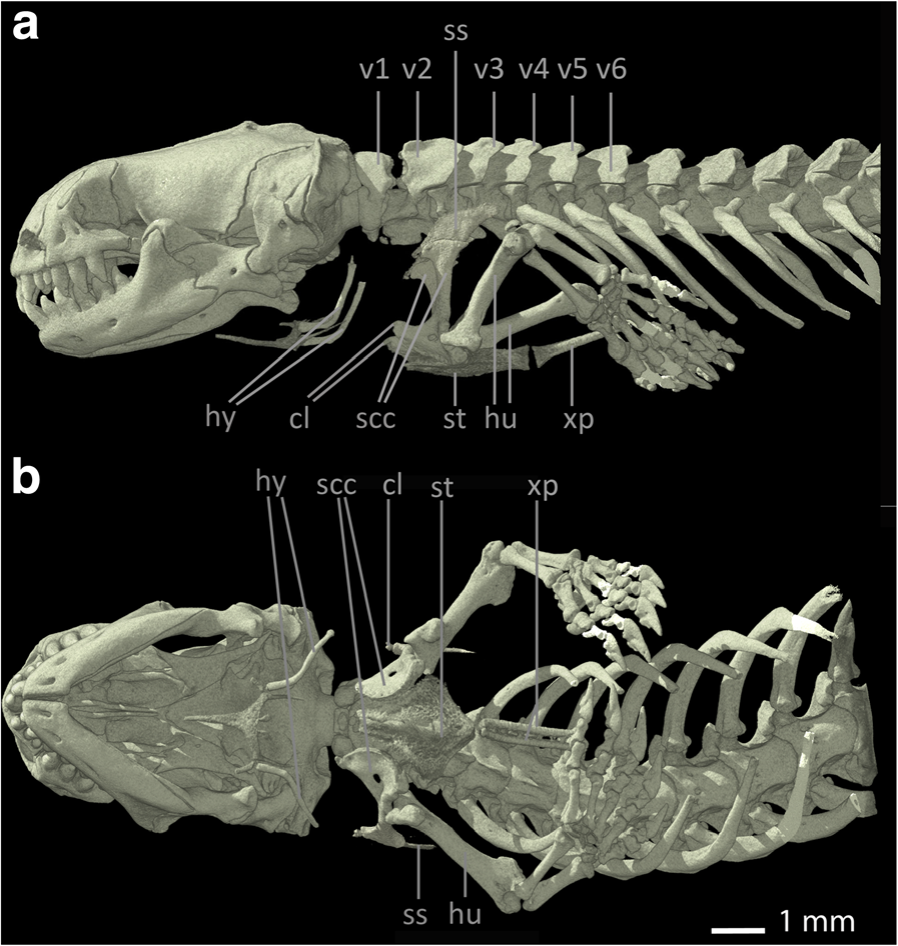
Skull, anterior part of the vertebral column and pectoral region of *Bipes biporus*. **a** lateral view, **b** ventral view. Abbreviations: cl: clavicles, hu: humerus, hy: hyoids, scc: scapulocoracoids, ss: suprascapula, st: sternum, v: vertebra, xp: xiphoid process

The shoulder girdle of *Bipes biporus* is the most complete among all amphisbaenians [8, 48] (see Fig. 1). It consists of a calcified, cartilaginous, rectangular **sternum (st)**, which is accompanied posteriorly by an extremely long, also calcified and cartilaginous **xiphoid process (xp)**. There is no connection between the sternum and the ribs. Anteriorly it is connected to the well-developed **scapulocoracoids (scc)** [8] which contain the proximally positioned coracoid foramina. The oval glenoid fossae of the scapulocoracoids articulate with the **humerus (hu)**. Distally, the scapulocoracoids articulate with the cartilaginous, but calcified, rectangular [30] **suprascapulae (ss)**. The suprascapulae are situated dorsally to the scapulocoracoids and possess an anteroventral process, which extends medial to the **clavicles (cl)**, but does not contact them. The latter are reduced, small, slender and rod-like bones that are connected to the shoulder girdle only via ligamentous connections to the sternum [8]. Also, they are situated relatively far away from the scapulocoracoids [49]. The shoulder girdle of *Bipes biporus* is positioned relatively close to the head at the level of the 3rd cervical vertebra [8]. For reasons of completeness and because it serves as insertion side for some muscles, the **hyoid (hy)** is also marked in all specimens.

Among amphisbaenians, Bipedidae are the only family with forelimbs [31] and possess an ossified stylo-, zeugo-, and autopodium [8]. The forelimbs are compact and robust [31] with well-developed digits [8, 50]. The humerus of *Bipes* is relatively slender and exhibits enlarged proximal and distal heads, as well as non-coalesced epiphyses [8]. The bone is twisted along its main axis, as it is characteristic for most Lepidosauria [8]. *Bipes biporus* possesses five fingers with three phalanges each (phalangeal formula 3, 3, 3, 3, 3) [8]. For a comparison of all pectoral girdle elements within the Amphisbaenian families see Table 1.

**Muscles of the shoulder girdle**

The identification of the muscles of the shoulder girdle of *Bipes biporus,* as well as those of the other amphisbaenian taxa, was made on the basis of their bony attachment sides, their overall topological arrangement, and generally by outgroup comparison. Because all respective muscle are described in detail for *Meroles cuneirostris* (see Additional file 2: Text S2), only general patterns are described in the following.

**Overview of present muscles**

**Fig. 2:**
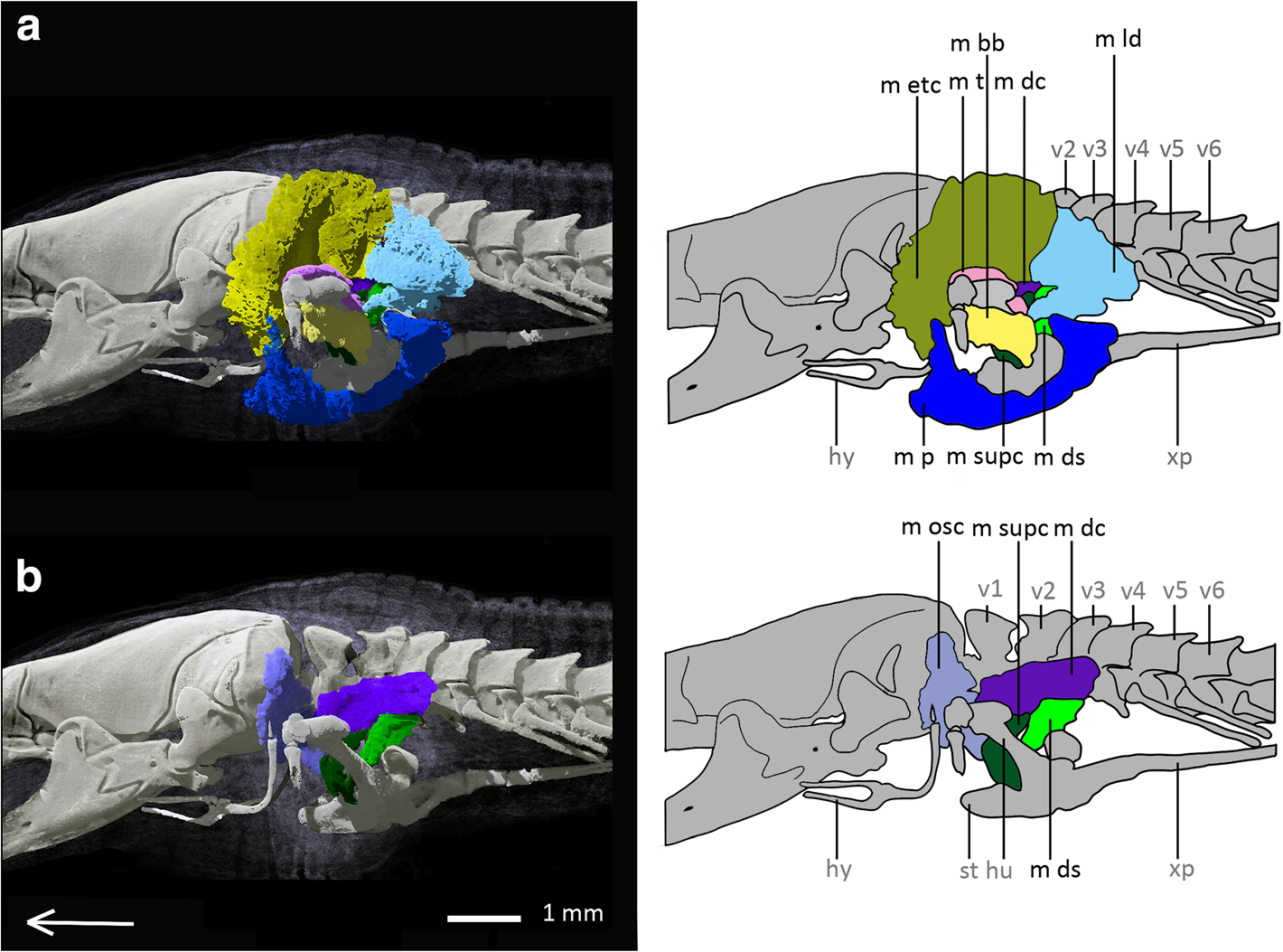
Superficial pectoral muscles of *Bipes biporus* in lateral view. **a** distal superficial pectoral muscles of *Bipes biporus*, **b** medial superficial pectoral muscles of *Bipes biporus*. Array shows anterior direction, abbreviations: hu: humerus, hy: hyoid, m bb: M. biceps brachii, m dc: M. deltoideus clavicularis, m ds: M. deltoideus scapularis, m etc: M. episternocleidomastoideus and M. trapezius-complex, m ld: M. latissimus dorsi, m osc: M. omohyoideus and M. sternohyoideus-complex, m p: M. pectoralis, m supc: M. supracoracoideus, m t: M. triceps, st: sternum, v: vertebra, xp: xiphoid process

**Fig. 3:**
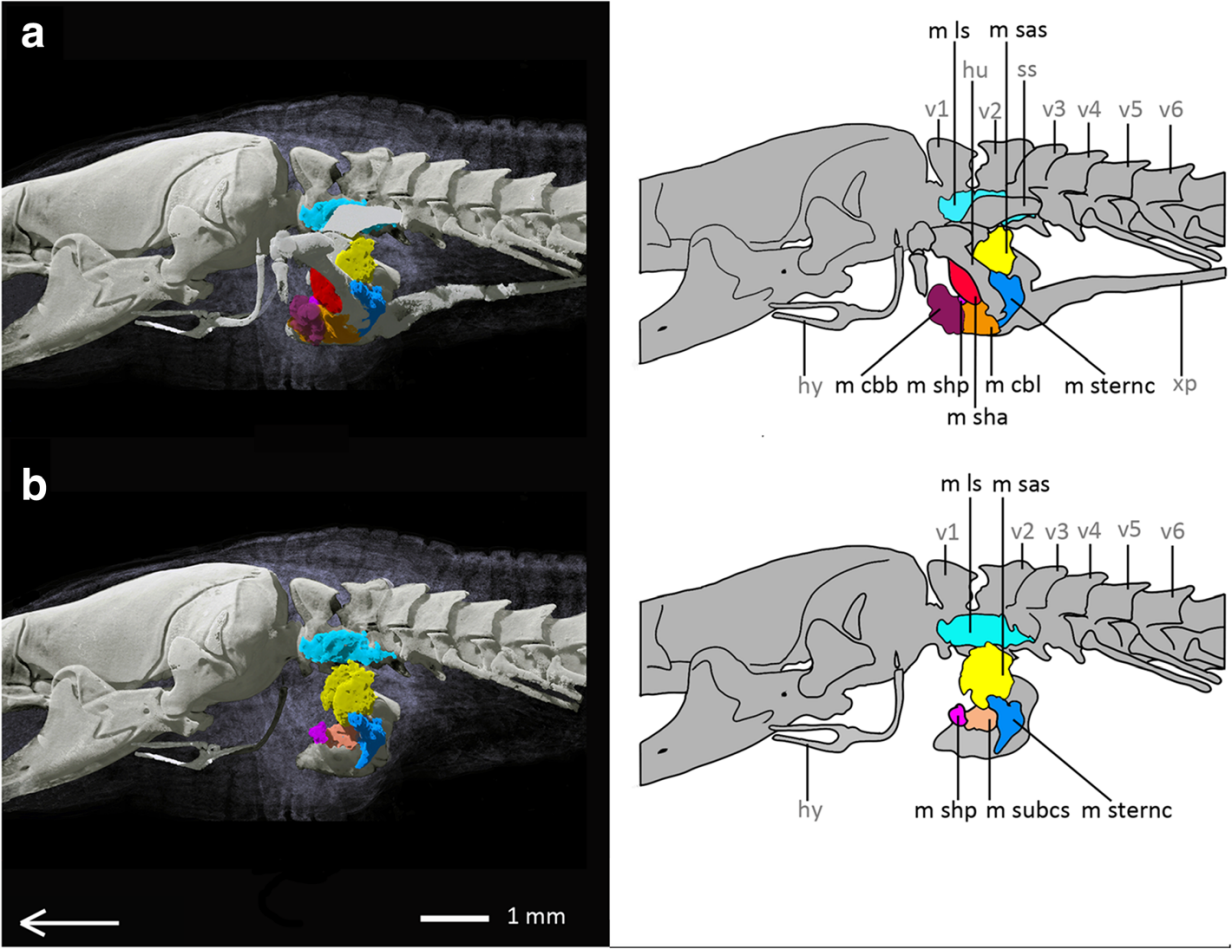
Subjacent pectoral muscles of *Bipes biporus* in lateral view. **a** Distal subjacent pectoral muscles of *Bipes biporus*, **b** medial subjacent pectoral muscles of *Bipes biporus*. Array shows anterior direction, abbreviations: hu: humerus, hy: hyoid, m cbb: M. coraco-brachialis brevis, m cbl: M. coraco-brachialis longus, m ls: M. levator scapulae, m sas: M. serratus anterior superficialis, m sha: M. scapulo-humeralis anterior, m shp: M. scapulo humeralis posterior, m sternc: M. sternocoracoideus, m subcs: M. subcoraco-scapularis, m supc: M. supracoracoideus, ss: suprascapulae, v: vertebra, xp: xiphoid process

**Fig. 4:**
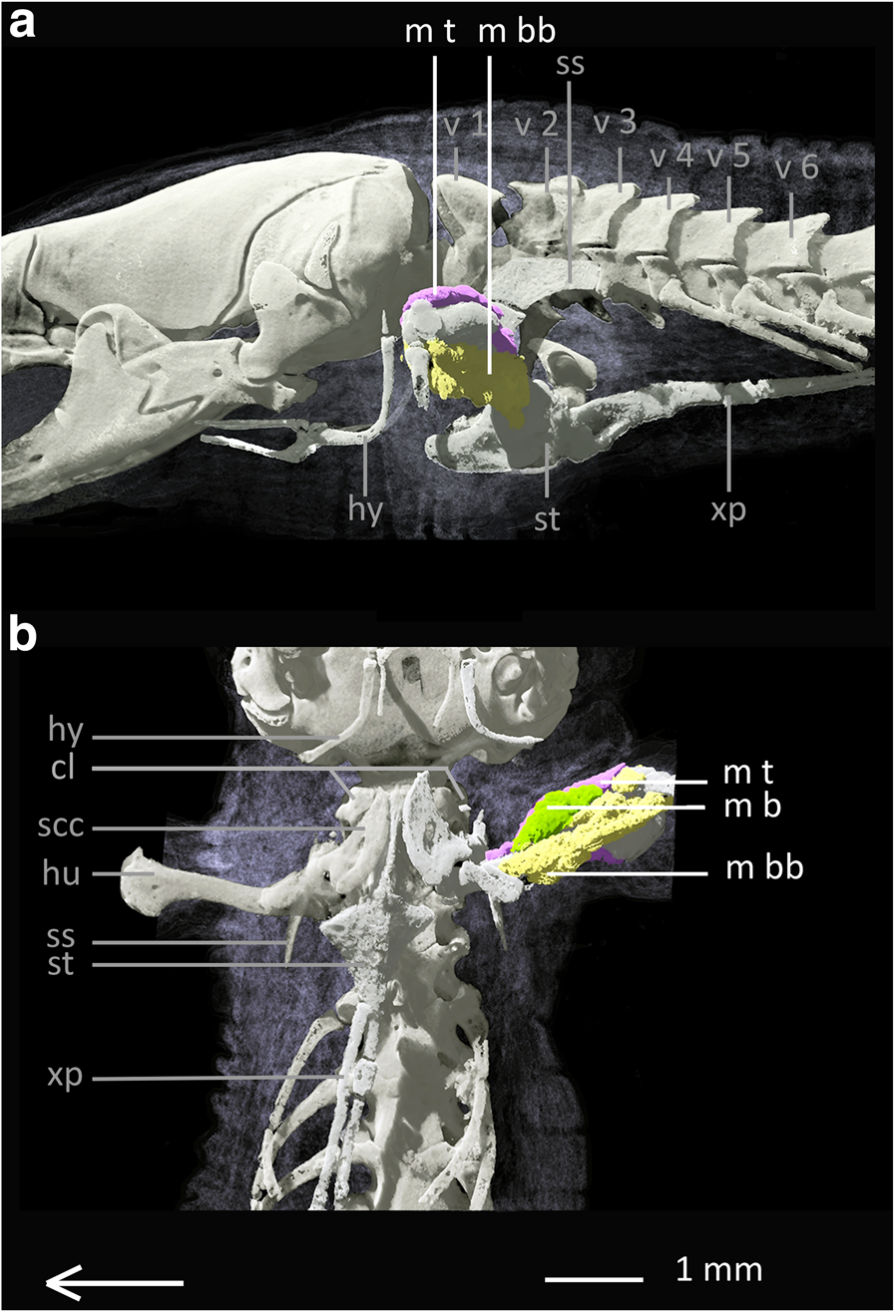
Muscles of the upper forelimb of *Bipes biporus*. **a** lateral view, **b** ventral view. Array shows anterior direction, abbreviations: cl: clavicles, hu: humerus, hy: hyoid, m b: M. brachialis m bb: M. biceps brachii, M t: M. triceps, scc: scapulocoracoid, ss: suprascapula, st: sternum, v: vertebra, xp: xiphoid process

*Bipes biporus* possesses nine superficial muscles (see Fig. 2), eight subjacent muscles (see Fig. 3) and three upper forelimb muscles (see Fig. 4). With the exception of the M. trapezius, which coalesces with the M. episternocleidomastoideus, and the M. omohyoideus and M. sternohyoideus, which also coalesce, all other muscles of the shoulder girdle are present as separate units.

**Superficial muscle layers**

The superficial pectoral muscles are positioned in two layers in all non-rhineurid amphisbaenians, an exterior (distal) and an interior (medial) layer.

The **M. latissimus dorsi (m ld)** occurs as a large, flat, dorsolateral muscle, similar to that of *Meroles cuneirostris,* but a little smaller relative to the other muscles*.* Posterior it is attached to the M. episternocleidomastoideus and M. trapezius-complex. It lies distally to the M. deltoideus clavicularis and arises from a dorsal layer of connective tissue as well as from the surface of the M. episternocleidomastoideus and M. trapezius-complex and runs ventrally to insert into the deltopectoral crest of the humerus.

The **M. episternocleidomastoideus and M. trapezius** are shown as separate muscles for *Bipes canalicatus* [34]*,* and also are divided into several portions. In our scans of *Bipes biporus,* M. episternocleidomastoideus and M. trapezius definitely form a large fibrous complex **(m etc)** with some fibres interacting, and no clear division can be made. Our observation is in accordance with Bolk et al. [51], state that M. episternocleidomastoideus and M. trapezius often are hard to separate from each other in squamates in general. In *Bipes biporus* this complex forms a relatively large muscle unit, which lies distally and anteriorly to the M. deltoideus clavicularis. The complex covers most of the M. deltoideus clavicularis and expands laterally and anteriorly in form of a flat layer that extends beyond the level of the M. deltoideus clavicularis. Similar to *Meroles cuneirostris*, in which both muscles are separate, the complex runs relatively far anterior, i.e. it covers the area from the second vertebra to the posterior part of the skull and covers also parts of the hyoid. The M. episternocleidomastoideus and M. trapezius complex arises from a dorsal layer of connective tissue near the skin and inserts into the cranium.

As mentioned above, **M. omohyoideus und M. sternohyoideus** form a complex **(m osc)** in *Bipes biporus*. This complex runs in a layer proximal to the M. episternocleidomastoideus and M. trapezius-complex. Similar to the latter, it arises from the upper third of the lateral body half from an inter-muscular area of connective tissue and runs to the hyoid. Together with the M. episternocleidomastoideus and M. trapezius-complex it is one of the most anteriorly positioned muscle units of the shoulder girdle.

The **M. deltoideus** **scapularis (m ds)** is a flat muscle that arises from the suprascapula and runs to the deltopectoral crest of the humerus, where it also inserts. It is smaller than the M. deltoideus scapularis of *Meroles cuneirostris* and much more posteriorly located.

Dorsal to M. deltoideus scapularis and M. serratus anterior the **M. deltoideus clavicularis (m dc)** is located, which runs distal to the suprascapula and extends beyond its level both anteriorly and posteriorly. It arises from the suprascapula and inserts into the scapulocoracoid as well as into the surface of the adjacent M. supracoracoideus anteriorly and M. deltoideus scapularis posteriorly. In comparison to the M. deltoideus clavicularis of *Meroles cuneirostris,* the M. deltoideus clavicularis of *Bipes biporus* exhibits no twist around its own axis. It is a long, flat muscle that is located not only anteroventrally, as in *Meroles cuneirostris*, but also dorsally to the M. deltoideus scapularis. It forms a muscle layer that is bordered distally by the M. episternocleidomastoideus and M. trapezius-complex as well as by the M. latissimus dorsi, and proximally by the suprascapula.

Anteroventrally to the M. deltoideus scapularis the **M. supracoracoideus (m supc)** is located. It is a long, flat muscle that runs between the attachment of the humerus to the shoulder girdle and the scapulocoracoid, from which it arises. Proximally it is strongly associated with the M. scapulo-humeralis anterior, from which it is also difficult to separate, at least in the contact zone. This observation is in accordance with Bolk et al. [51], who state that the latter two muscles are sometimes fused in lizards. Also in *Meroles cuneirostris* there is a strong association between the M. supracoracoideus and the M. scapulo-humeralis anterior, but they can be much better separated from each other than in *Bipes biporus*. Here, the M. supracoracoideus runs with a twist around its own axis in direction of the deltopectoral crest of the humerus, where it also inserts.

The flat **M. pectoralis** **(m p)** consists, like in *Meroles cuneirostris*, of diverse layers, which partly overlap each other. As a superficial muscle it arises from a layer of connective tissue between skin and sternum and runs across the sternum and inserts into the deltopectoral crest of the humerus. Both anterior and posterior to the humerus it extends far dorsally and covers the M. episternocleidomastoideus and M. trapezius- complex anteriorly. Posteriorly it is connected to the M. latissimus dorsi. It runs distally to the M. coraco-brachialis longus.

**Subjacent muscle layers**

The subjacent pectoral muscles are also positioned in two layers in all non-rhineurid amphisbaenians, an exterior (distal) and an interior (medial) layer.

Proximal to the M. deltoideus scapularis the **M. serratus anterior superficialis (m sas)** is located. It is a flat muscle that runs proximally from the suprascapula to the scapulocoracoid and inserts into the deltopectoral crest of the humerus.

The **M. scapulo-humeralis anterior (m sha)** runs proximal to the M. supracoracoideus and ventral to the M. deltoideus clavicularis. It arises from both the clavicle and the scapulocoracoid, runs directly along the scapulocoracoid and inserts into the deltopectoral crest of the humerus.

Relative to the latter muscle, the **M. scapulo-humeralis posterior (m shp)** is located proximolaterally, whereas both are strongly associated with each other. The origin of the M. scapula-humeralis posterior lies on the scapulocoracoid and its insertion is found on the surface of the adjacent muscles, i. e. M. subcoraco-scapularis, M. coraco-brachialis brevis and M. coraco-brachialis longus.

Proximal to the suprascapula, the **M. levator scapulae (m ls)** is located, which arises from the lateral process of the atlas in the same way as in Lacertidae [52]. It runs proximal to the suprascapula where it also inserts. The M. levator scapulae is of a robust, elongated shape and is covered by the M. deltoideus clavicularis and the M. episternocleidomastoideus and M. trapezius-complex, being one of the most proximally situated muscles of the shoulder girdle. It runs in a medial layer dorsal to the M. serratus anterior superficialis, which differs from its position in *Meroles cuneirostris*, in which it is located anterior to the M. serratus anterior superficialis.

The **M. coraco-brachialis brevis (m cbb)** is located below the articulation of the humerus to the shoulder girdle. It is a flat, bulky muscle that runs ventrally to the M. supracoracoideus and M. scapulo-humeralis anterior, as well as the M. omohyoideus and the M. sternohyoideus-complex. It arises from a layer of inter-muscular connective tissue and inserts into another layer of connective tissue as well as into the surface of the adjacent M. coraco-brachialis longus.

Proximally, deep below the M. coraco-brachialis brevis, the **M. coraco-brachialis longus (m cbl)** is located. It is larger than the former muscle and arises from the scapulocoracoid, inserting into the deltopectoral crest of the humerus, where it is positioned adjacent to the M. biceps brachii.

One of the most proximally situated muscles of *Bipes biporus* is the **M. subcoraco-scapularis (m subcs)**, an aggregation of M. subcoracoideus and M. subscapularis [51], which runs proximal to the scapulocoracoid. The muscle is much smaller compared to the M. subcoraco-scapularis of *Meroles cuneirostris*. It arises from a ventrally positioned layer of connective tissue inside the body and inserts into the deltopectoral crest of the humerus and is positioned ventrally to the M. serratus anterior superficialis.

Laterally to the M. subcoraco-scapularis the **M. sternocoracoideus (m sternc)** is found, which is relatively large compared to *Blanus* and *Trogonophis*. It arises in a layer of connective tissue near the sternum and runs around the deltopectoral crest of the humerus, where it inserts.

**Muscles of the upper forelimb**

The muscles of the upper forelimb are the **M. biceps brachii (m bb)**, the **M. brachialis** **(m b)** and the **M. triceps (m t).** They all run along the humerus in a similar way and with identical attachment sites as in *Meroles cuneirostris* (see Additional file 2: Text S2 for details).

##### Blanidae (*Blanus strauchi*)

**Bones of the shoulder girdle**

**Fig. 5:**
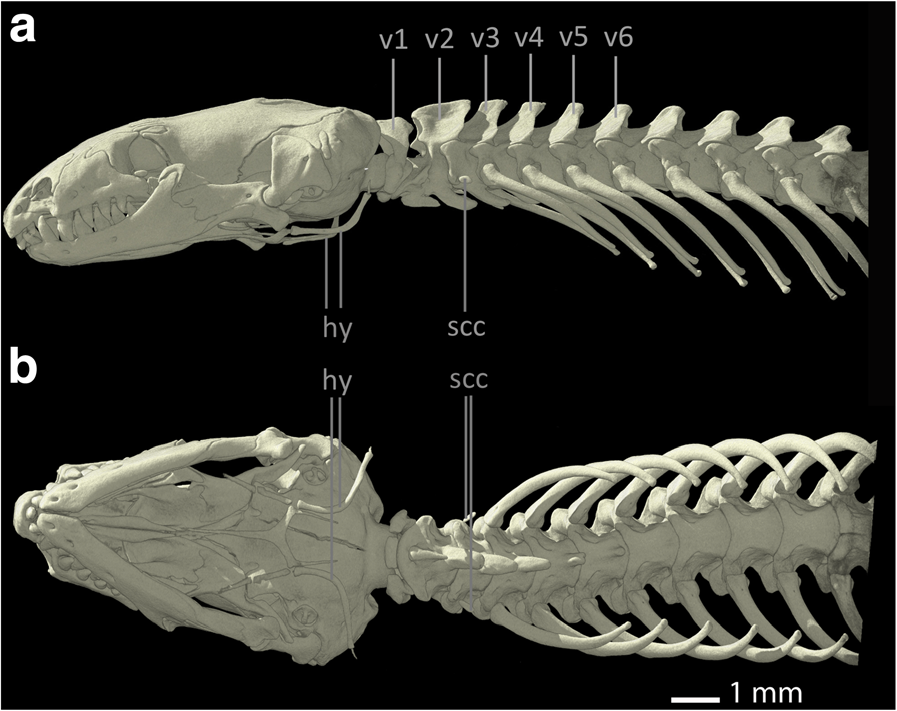
Skull, anterior part of the vertebral column and pectoral region of *Blanus strauchi*. **a** lateral view, **b** ventral view. Abbreviations: hy: hyoids, scc: scapulocoracoids, v: vertebra

Several authors [8, 30, 53] state that *Blanus cinereus* retains vestiges of the shoulder girdle, i.e. a cartilaginous **sternum**, rodlike, slender, and ossified **scapulocoracoids** with cartilaginous tips and small bony **clavicles** [8]. In our scans of *Blanus strauchi*, small bony scapulocoracoids can be found, but they are much smaller than those described for *Blanus cinereus*, being positioned at the level of the third to the fourth vertebra (see Fig. 5). Whereas we assume that there is also a cartilaginous sternum, we were unable to verify this conjecture because cartilage cannot be visualized when staining with IKI. Also, in our scan of *Blanus strauchi* no clavicles were found, but in comparison to scans of additional specimens of *Blanus strauchi* (data not shown) it is apparent that there is variation in both the presence of vestigial clavicles and in the size of the scapulocoracoids: In some specimens the clavicles are absent; in other specimen the scapulocoracoids are smaller than described in the literature. This variation most likely explains the absence of clavicles and the reduced size of the scapulocoracoids in our stained specimen.

**Muscles of the shoulder girdle**

**Overview of present muscles**

**Fig. 6:**
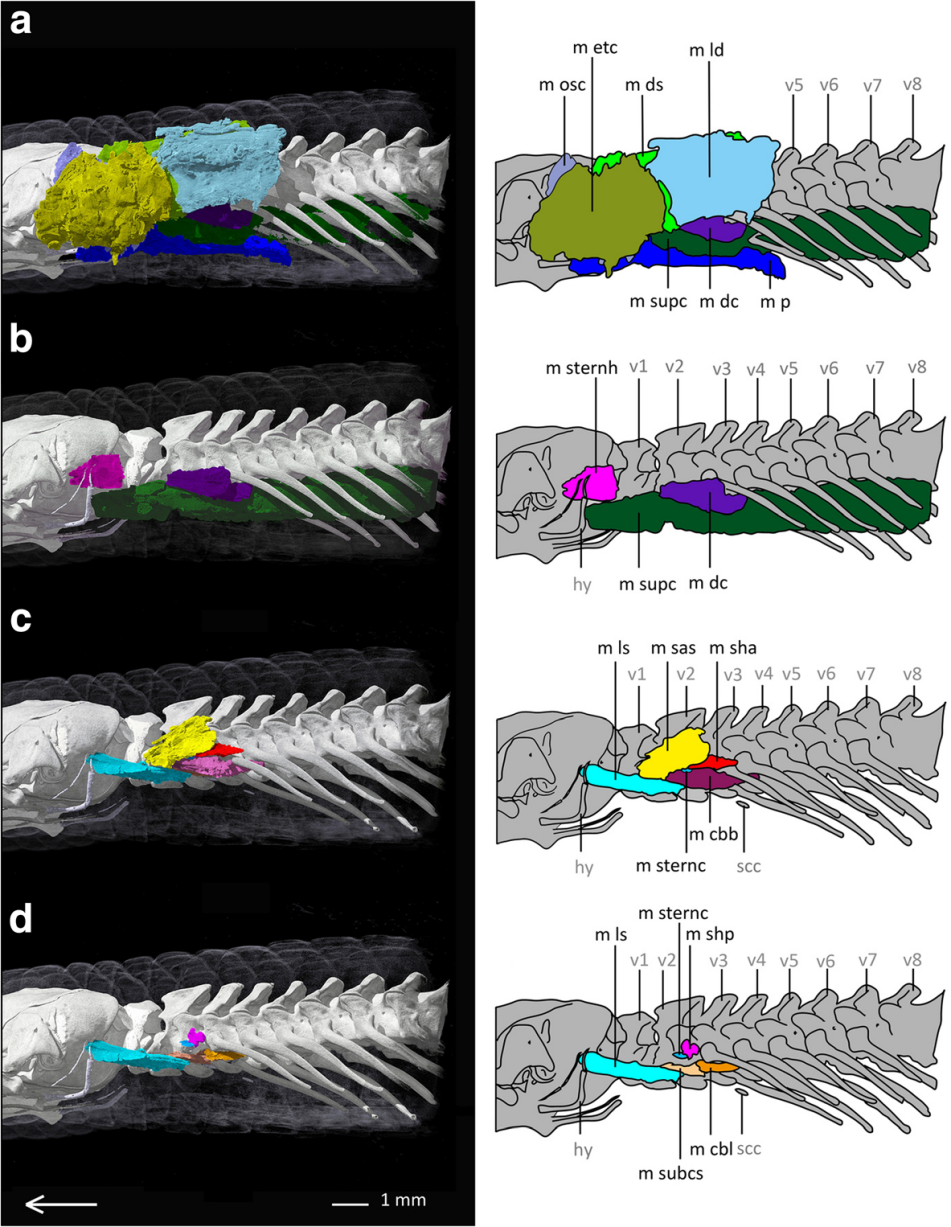
Pectoral muscles of *Blanus strauchi* in lateral view. **a** distal superficial pectoral muscles of *Blanus strauchi*, **b** medial superficial pectoral muscles of *Blanus strauchi*, **c** distal subjacent pectoral muscles of *Blanus strauchi*, **d** medial subjacent pectoral muscles of *Blanus strauchi*. Array shows anterior direction, abbreviations: hy: hyoid, m cbb: M. coraco-brachialis brevis, m cbl: M. coraco-brachialis longus, m dc: M. deltoideus clavicularis, m ds: M. deltoideus scapularis, m etc: M. episternocleidomastoideus and M. trapezius-complex, m ld: M. latissimus dorsi, m ls: M. levator scapulae, m osc: M. omohyoideus and M. sternohyoideus-complex, m p: M. pectoralis, m sas: M. serratus anterior superficialis, m sha: M. scapulo-humeralis anterior, m shp: M. scapulo-humeralis posterior, m sternc: M. sternocoracoideus, m sternh: M. sternohyoideus, m subcs: M. subcoraco-scapularis, m supc: M. supracoracoideus, scc: scapulocoracoid, v: vertebra

In *Blanus strauchi* all muscles of the shoulder girdle are present, consisting of nine superficial (see Fig. 6a, b) and eight subjacent muscles (see Fig. 6c, d). M. episternocleidomastoideus and M. trapezius form a complex similar to *Bipes biporus*, but M. omohyoideus and M. sternohyoideus are present as separate muscles, like in *Meroles cuneirostris*. Due to the reduction of the forelimbs, *Blanus strauchi* has no upper arm muscles.

**Superficial muscle layers**

The **M. latissimus dorsi** of *Blanus strauchi* is a relatively large, thin muscle layer covering the posterolateral side of the body. It arises near the skin from a dorsal layer of connective tissue and runs ventrally where it inserts into another layer of connective tissue near the skin; in the posterior region it runs further ventrally than in the anterior region. The muscle is similar to the M. latissimus dorsi of *Meroles cuneirostris* and *Bipes biporus*, but it is associated with the M. deltoideus scapularis in its posterior region, which is not the case in the other taxa examined in this study.

The **M. episternocleidomastoideus and M. trapezius** form a large complex, which is almost as large as the M. latissimus dorsi. It arises from connective tissue situated between the M. deltoideus scapularis and the outer body wall. There is no association between the M. latissimus dorsi and the M. episternocleidomastoideus and M. trapezius-complex. The M. episternocleidomastoideus and M. trapezius-complex runs with parallel fibres in a dorsoventral direction, passing the hyoid and inserting near the cranium. It is the most anteriorly positioned shoulder muscle in *Blanus strauchi*. In its morphology it is similar to the M. episternocleidomastoideus and M. trapezius-complex of *Bipes biporus*.

The **M. omohyoideus** is a relatively large, single muscle running tubular through the specimen. In lateral view it is a trapezoid-shaped muscle mass. It is located proximal to the M. episternocleidomastoideus and M. trapezius-complex, by which it is almost fully covered except for the most anterodorsal part. The muscle originates in the dorsal region from a layer of connective tissue situated between the M. deltoideus scapularis and the M. episternocleidomastoideus and M. trapezius-complex, and runs with parallel fibres to the ventral body side as well as to the cranium. It inserts into a layer of connective tissue near the M. pectoralis, into the hyoid and also near the cranium. Posteriorly it is slightly associated with the M. episternocleidomastoideus and M. trapezius-complex.

The **M. sternohyoideus** is a trapezoid muscle mass proximal to the M. omohyoideus. It is surrounded by the M. deltoideus scapularis and the M. episternocleidomastoideus and M. trapezius-complex distally, and by the M. levator scapulae and the cranium proximally. It arises from a layer of connective tissue near the M. deltoideus scapularis and runs in anterior direction, passing the hyoid and inserting into the cranium at the level of the latter bone.

The **M. deltoideus** **scapularis** appears as a large, massive muscle that is partly covered by the M. latissimus dorsi posteriorly and the M. episternocleidomastoideus and M. trapezius-complex as well as the M. omohyoideus anteriorly. It is slightly associated with the M. deltoideus clavicularis in the latter’s posterior region. It arises dorsally from a sheet of connective tissue beneath the skin and runs with parallel fibres to the ventral body side. It inserts into a muscle strand near the cranium at the level of the braincase near the inner ear. The muscle is similar to the M. deltoideus scapularis of *Meroles cuneirostris* and *Bipes biporus*.

The **M deltoideus clavicularis** is located ventrally to the M. deltoideus scapularis. It is smaller than the latter and partly overlain by it. The M. deltoideus clavicularis arises from an undifferentiated muscle near the first rib and is strongly associated with the M. coraco-brachialis brevis, which is located proximally. It runs anteriorly with parallel fibres and inserts into a layer of connective tissue between the M. serratus anterior superficialis and the M. deltoideus scapularis.

The **M. supracoracoideus** is located ventrally to the M. deltoideus clavicularis and arises near the ribs. It is a very large and elongated muscle mass running anteriorly along the ventral side of the body. It is partly covered by the M. pectoralis and inserts into the cranium. The M. supracoracoideus of *Blanus strauchi* is much larger than in all other examined species.

The **M. pectoralis** closes up the muscle mass on the ventral side, like in all other examined taxa. It arises from the hyoid and runs posteriorly to insert into a layer of connective tissue near the skin. It consists of several thin, partly overlapping layers. Halfway it is associated with the M. supracoracoideus, which also runs ventrally. Anteriorly it meets the M. episternocleidomastoideus and M. trapezius-complex, but is not associated with the latter. In contrast to *Bipes biporus*, the muscle is posteriorly not in contact with the M. latissimus dorsi.

**Subjacent muscle layers**

The **M. serratus anterior superficialis** is located proximally to the M. deltoideus scapularis and the M. deltoideus clavicularis. It arises from the surface of the M. deltoideus scapularis, with which is also strongly associated. The muscle is very fibrous and consists of two parts that are strongly associated with each other and therefore hard to separate. It runs anteriorly and inserts into a layer of connective tissue near the M. levator scapulae and the vertebral column.

The **M. scapulo-humeralis anterior** is located proximally to the M. serratus anterior superficialis, M. deltoideus scapularis, M. deltoideus clavicularis and M. latissimus dorsi. It appears as a relatively small, elongated muscle that arises at the level of the first ribs and runs anteriorly. It passes the scapulocoracoid and inserts into a layer of connective tissue between the M. serratus anterior superficialis and the vertebral column.

The **M. scapulo-humeralis posterior** is located proximally to the M. scapulo-humeralis anterior. It is a small muscle that is entirely covered by the M. scapulo-humeralis anterior. It arises from the scapulocoracoid, runs anteriorly and inserts into the vertebral column near the end of the second vertebra. Its morphology is similar to that of *Bipes biporus*.

The **M. levator scapulae** shows a cranial origin near the hyoid. It runs posteriorly with a slightly ventral drift and inserts into the M. supracoracoideus. It is an elongated, bulky muscle that originates from the cranium at the level of the hyoid. It is located anteriorly to the M. serratus anterior superficialis in lateral view.

The **M. coraco-brachialis brevis** is an elongated, bulky muscle mass strongly associated with the M. deltoideus clavicularis. It arises between the first and the second rib, runs anteriorly and inserts into a layer of connective tissue next to the vertebral column. It passes the scapulocoracoid and is also connected to the latter.

Proximally to the M. supracoracoideus, M. coraco-brachialis brevis and M. deltoideus clavicularis the **M. coraco-brachialis longus** is located. It is a thin, elongated muscle running on the ventral side of the body along the ribs. It arises from the M. supracoracoideus and runs anteriorly to insert into the vertebral column near the M. subcoraco-scapularis, which is a pattern that is different from all other examined taxa.

The **M. subcoraco-scapularis** arises from, and runs along the vertebral column. It is the most proximally situated muscle and of an elongated morphology. The muscle runs for one third of its course next to the M. coraco-brachialis longus and in its most anterior third next to the M. levator scapulae. It generally runs anteriorly, passing the scapulocoracoid, and inserts into the vertebral column.

The **M. sternocoracoideus** is the smallest muscle in *Blanus strauchi*, having an oval to rotund shape. It arises from the surface of the M. coraco-brachialis brevis and runs anteriorly along the scapulocoracoid. The muscle inserts into the M. scapulo-humeralis anterior and the M. coraco-brachialis brevis.

##### Trogonophidae (*Trogonophis wiegmanni*)

**Bones of the shoulder girdle**

**Fig. 7:**
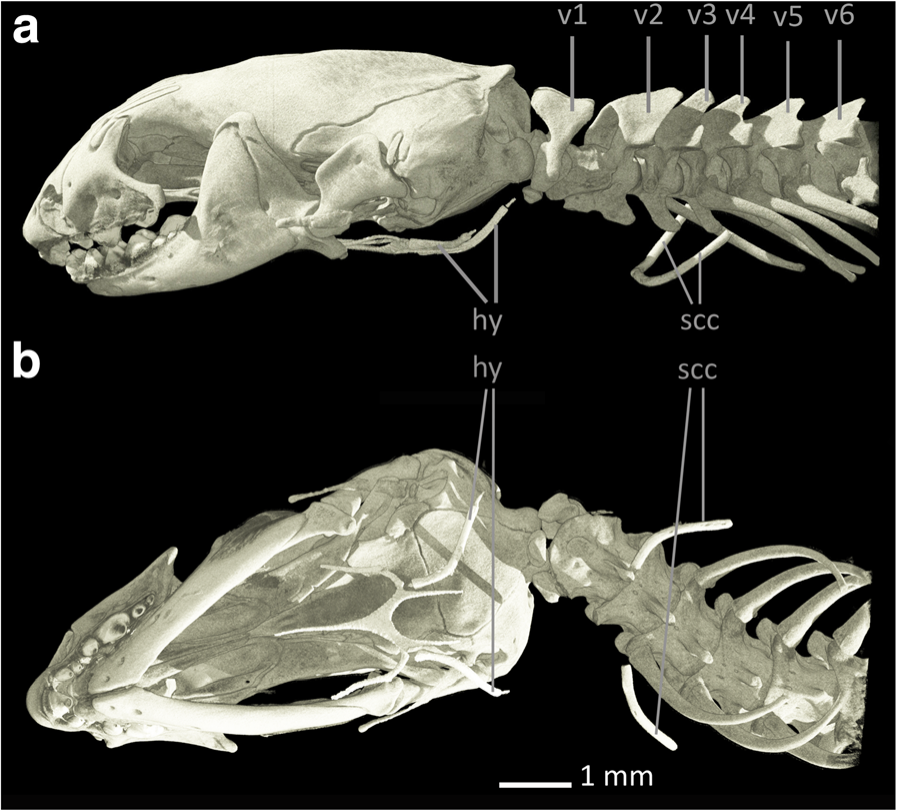
Skull, anterior part of the vertebral column and pectoral region of *Trogonophis wiegmanni*. **a** lateral view, **b** ventral view. Abbreviations: hy: hyoids, scc: scapulocoracoids, v: vertebra

The shoulder girdle of *Trogonophis wiegmanni* has already been described by several authors [8, 30, 33, 53–55]. Although Zangerl [30] assumed that there must be cartilaginous and bony vestiges of the shoulder girdle in *Trogonophis wiegmanni*, he did not find any in his cleared and stained specimens, and therefore reasoned that they must have been lost during maceration. All other of the above authors found indeed remnants of the shoulder girdle in *Trogonophis wiegmanni*, which appeared reduced in comparison to *Bipes biporus*, consisting of only three elements [55]. The anteroposteriorly narrow and mediolaterally elongated, cartilaginous **sternum** [8] is stirrup-shaped [55] and accompanied on either side by the bony, rod-shaped **scapulocoracoids** [55] (see Fig. 7). Both scapulocoracoids are loosely linked to the sternum by ligaments of the M. sternocoracoideus [55] and posteriorly bend towards their connection to the sternum [8]. While the sternum is located immediately beneath the skin, both scapulocoracoids seem to be embedded by surrounding musculature [55] and are thickened at their endings. The scapulocoracoids possess cartilaginous distal tips [8] and are bent medially, while their proximal endings are expanded, creating a larger surface for the adhesion of muscle fibres [55]. The sternum is not linked to the ribs, and the clavicles are absent [53]. In our μ-CT scan the scapulocoracoids are located at the level of the third to fourth vertebra (i.e. first to second cervical vertebra following the atlas-axis-complex) and the sternum is not visible because of its cartilaginous character. The vertebra following the scapulocoracoids bears the first ribs. The relatively anterior position of the scapulocoracoids was already mentioned by Fürbringer [53], stating that the position of the shoulder girdle rudiments of *Trogonophis wiegmanni* could be found “at the level of the third to fourth vertebra” [53].

**Muscles of the shoulder girdle**

**Overview of present muscles**

**Fig. 8:**
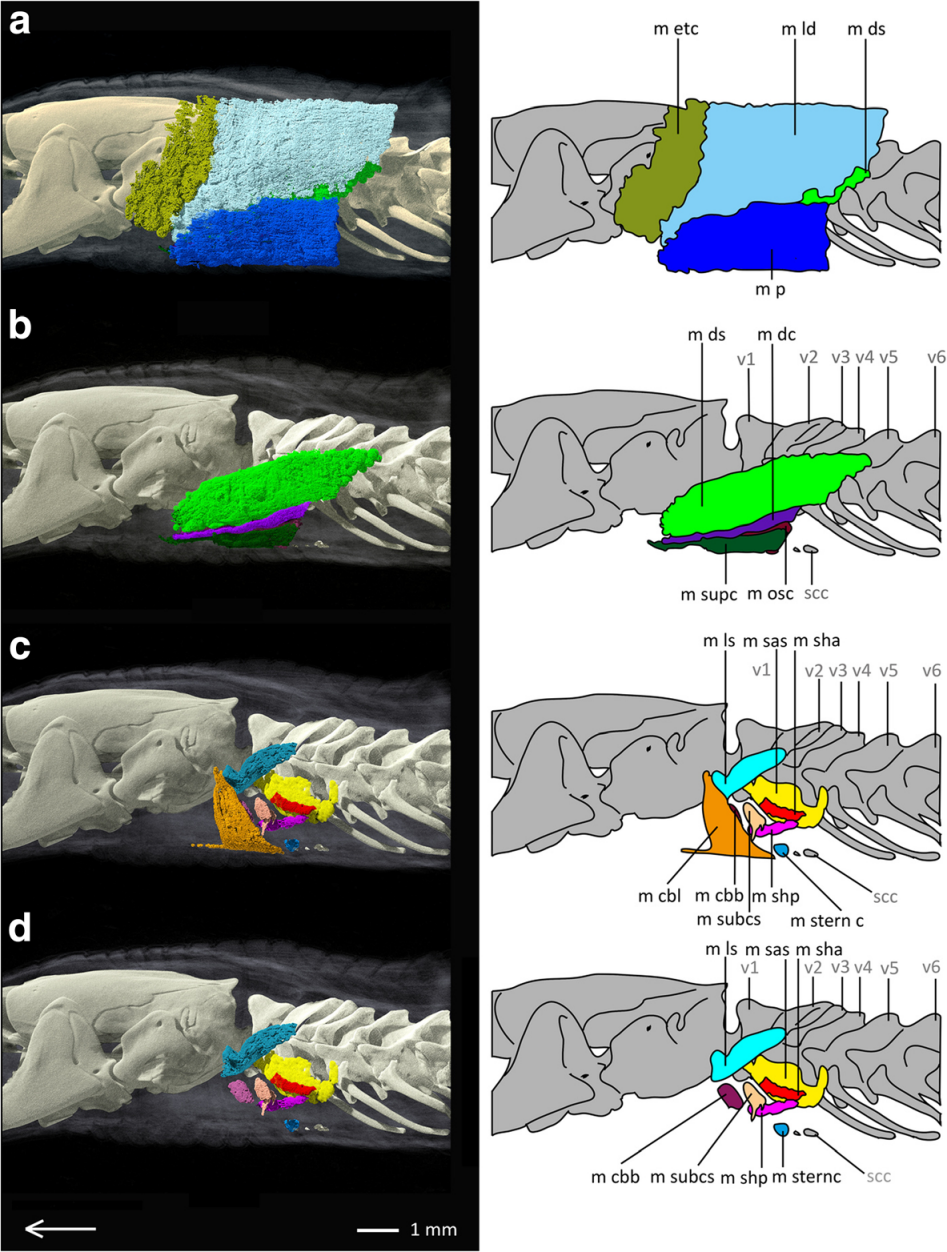
Pectoral muscles of *Trogonophis wiegmanni* in lateral view. **a** distal superficial pectoral muscles of *Trogonophis wiegmanni*, **b** medial superficial pectoral muscles of *Trogonophis wiegmanni*, **c** distal subjacent pectoral muscles of *Trogonophis wiegmanni*, **d** medial subjacent pectoral muscles of *Trogonophis wiegmanni*. Array shows anterior direction, abbreviations: m cbb: M. coraco-brachialis brevis, m cbl: M. coraco-brachialis longus, m dc: M. deltoideus clavicularis, m ds: M. deltoideus scapularis, m etc: M. episternocleidomastoideus and M. trapezius-complex, m ld: M. latissimus dorsi, m ls: M. levator scapulae, m osc: M. omohyoideus and M. sternohyoideus-complex, m p: M. pectoralis, m sas: M. serratus anterior superficialis, m sha: M. scapulo-humeralis anterior, m shp: M. scapulo-humeralis posterior, m sternc: M. sternocoracoideus, m subcs: M. subcoraco-scapularis, m supc: M. supracoracoideus, scc: scapulocoracoid, v: vertebra

All nine superficial (see Fig. 8a, b) and eight subjacent muscles (see Fig. 8c, d) of the shoulder girdle of *Trogonophis wiegmanni* are present as single muscles, except for the M. episternocleidomastoideus and the M. trapezius, and the M. omohyoideus and the M. sternohyoideus, which are again fused to complexes. *Trogonophis wiegmanni* possesses no forelimbs and therefore no humerus. Also muscles belonging to the forearm (i.e. M. biceps brachii, M. brachialis and M. triceps) are absent.

**Superficial muscle layers**

The **M. latissimus dorsi** is a prominent, flat, dorsolaterally running muscle, which is larger than in *Meroles cuneirostris*, *Bipes biporus* and *Blanus strauchi*. It arises dorsally from a layer of connective tissue and runs ventrally, i.e. directly next to the similarly flat complex of M. episternocleidomastoideus and M. trapezius, which results in the absence of any sharp division between the two muscles units. It inserts into another ventral layer of connective tissue near the skin.

The **M. episternocleidomastoideus and M. trapezius-complex** represents a superficial muscle mass that arises from the vertebral column, runs anteroventrally, and inserts near the cranium. Furthermore, the M. episternocleidomastoideus and M. trapezius-complex is the most anteriorly situated muscle mass, similar to *Blanus strauchi*, but is smaller than in the latter. The M. latissimus dorsi and the M. episternocleidomastoideus and M. trapezius-complex of *Trogonophis wiegmanni* run far ventrally, similar to *Bipes biporus*. Another important difference is that the M. latissimus dorsi and the M. episternocleidomastoideus and M. trapezius-complex are located directly next to each other in *Trogonophis wiegmanni,* whereas in *Blanus strauchi* there is no association between the M. latissimus dorsi and the M. episternocleidomastoideus and M. trapezius complex.

Also **M. omohyoideus and M. sternohyoideus** form a complex, whereas it seems that the M. omohyoideus adapts to the course of the M. sternohyoideus, because the complex arises from the scapulocoracoid and runs anteriorly to insert into the hyoid. In contrast, the course of the M. omohyoideus and M. sternohyoideus-complex is dominated by the M. omohyoideus in the other examined taxa. The M. omohyoideus has generally been described to arise from both the clavicles and the interclavicle in squamates [55], whereas the M. sternohyoideus arises from the sternum and the medial end of the clavicle [56]. In *Trogonophis wiegmanni* the clavicles are reduced, whereas the scapulocoracoids and a cartilaginous sternum are still present (see above). The muscle complex arises from the scapulocoracoid, which is why we assumed that the M. sternohyoideus is the more dominant part within this complex. Because the complex consists of two long, parallel running muscles, one can assume that there was a fusion with the M. omohyoideus. A differentiation between the M. omohyoideus and the M. sternohyoideus is therefore not possible.

The massive and large **M. deltoideus scapularis** is dorsally overlain by the M. latissimus dorsi. The muscle shows a more strand-like morphology than in *Blanus strauchi*. Also, the fibre orientation of the M. deltoideus scapularis of *Trogonophis wiegmanni* is clearly different from the dorsoventral orientation seen in *Blaunus strauchi.* In *Bipes biporus* the M. deltoideus scapularis arises from the suprascapula, whereas in *Trogonophis wiegmanni* it arises from the surface of the M. levator scapulae and M. longissimus. The M. longissimus is a skeletal muscle of the back and runs in direction to the head. It belongs to the epaxial ancestral muscles, which are situated dorsally to the transverse processes of the vertebrae [56]. The origin of the M. deltoideus scapularis along the M. longissimus is located far posteriorly in the specimen, even beyond the level of the scapulocoracoids. Because of the reduction of the scapula, which was the former area of origin for the M. deltoideus scapularis, the muscle now originates from non-skeletal elements. The M. deltoideus scapularis runs craniodorsally and inserts into an area of connective tissue near the cranium. The M. deltoideus scapularis is covered by the M. latissimus dorsi dorsally and by the M. pectoralis ventrally.

Ventrally to the M. deltoideus scapularis the narrow, elongated **M. deltoideus clavicularis** is found. It arises from the scapulocoracoid and joins the whole course of the M. deltoideus scapularis with parallel-running fibres, until it also ends in the already mentioned area of connective tissue near the cranium. It inserts also in parts into the ventrally running M. supracoracoideus. The M. deltoideus clavicularis also is covered by the M. latissimus dorsi dorsally and by the M. pectoralis ventrally.

The **M. supracoracoideus** is located ventrally to the M. deltoideus clavicularis that arises from the scapulocoracoid, joins the course of the M. deltoideus scapularis and the M. deltoideus clavicularis, and inserts near the cranium, but a bit further anteriorly than the latter two. The M. deltoideus scapularis, the M. deltoideus clavicularis and the M. supracoracoideus all diverge strongly from their morphology in *Meroles cuneirostris* and *Bipes biporus*. While they represent a fan-shaped structure in *Meroles cuneirostris* and *Bipes biporus*, they appear as elongated muscle strands in *Trogonophis wiegmanni*.

The **M. pectoralis** completes the shoulder muscle complex ventrally. It arises from the scapulocoracoid near the sternum and runs in form of several thin, partly overlapping layers across the scapulocoracoid anteriorly. The muscle reaches far dorsally, even more than in *Blanus strauchi*. In its dorsal region it is in contact with the M. latissimus dorsi, which is slightly overlain in the area where both muscles meet. The M. pectoralis inserts into a layer of connective tissue near the skin.

**Subjacent muscle layers**

The **M. serratus anterior superficialis** is located proximally to the M. deltoideus scapularis, by which it is also almost completely covered. In its morphology it is quite similar to that of *Blanus strauchi*. It arises posterior to the origin of the M. levator scapulae on the first vertebra and runs caudally, where it inserts near the scapulocoracoid on the surface of the M. scapulo-humeralis anterior and the M. scapulo-humeralis posterior. As in *Meroles cuneirostris*, fibres run parallel with a posteroventral to anterodorsal propensity.

Lateral to the M. serratus anterior superficialis the **M. scapulo-humeralis anterior** is located, representing a relatively small, elongated muscle, which is covered distally by the M. deltoideus scapularis and the M. deltoideus clavicularis. It arises from the scapulocoracoid and runs anteriorly to the M. serratus anterior superficialis, where it also inserts. The muscle is similar to the M. scapulo-humeralis anterior of *Blanus strauchi*, whereas in *Trogonophis wiegmanni* it runs more distally.

Ventrally to the M. serratus anterior superficialis the elongated **M. scapulo-humeralis posterior** is situated, which is completely covered by the distally running M. deltoideus clavicularis. It arises near the scapulocoracoid and runs anteroproximally into the center of the body to insert into an area of connective tissue near the M. coraco-brachialis brevis.

The **M. levator scapulae** belongs to the most proximally situated muscles and arises from an inter-muscular area near the lower level of the cranium. Shortly behind its origin it makes a half-turn around its own axis and runs posteriorly with a dorsal drift, inserting into the surface of the M. longissimus. With respect to its position relative to the cranium, the M. levator scapulae of *Meroles cuneirostris* and *Trogonophis wiegmanni* superficially show similarities, although the insertion areas are different. While the M. levator scapulae of *Meroles cuneirostris* inserts into the scapula and sternum, in *Trogonophis wiegmanni* this insertion is into the surface of the M. longissimus. The insertion into another muscle is similar to the situation in *Blanus strauchi*, in which the M. levator scapulae inserts into M. supracoracoideus (see above). The M. levator scapulae is also similar to that of *Blanus strauchi* with regards to its shape and cranial origin near the hyoid.

The **M. coraco-brachialis brevis** strongly differs in morphology from *Meroles cuneirostris* and the other examined amphisbaenians. In *Meroles cuneirostris* it is a relatively large and spacious muscle, whereas in *Trogonophis wiegmanni* it is rather small and oval in shape. The muscle is situated relatively medially inside the body and is not in contact with any other muscle except for the M. coraco-brachialis longus. It arises and inserts into connective tissue, close to the M. scapulo-humeralis posterior.

The **M. coraco-brachialis longus** arises in the ventral region of the body near the skin. It shows a skewed anterodorsal course and is of trapezoid shape, whereas its apex points anteriorly. It inserts into a layer of connective tissue near the origin of the M. levator scapulae. In *Trogonophis wiegmanni* this muscle is large in comparison to the other examined species.

The **M. subcoraco-scapularis** is a combination of the M. subcoracoideus and M. subscapularis [57]. In *Trogonophis wiegmanni* there is a visible border between these two units. The larger, bulkier of the two originates from an area of connective tissue near the M. deltoideus clavicularis and runs distally from there to another intramuscular bundle of connective tissue inside the body near the M. serratus anterior superficialis. The other, smaller and more elongated part is situated rather proximally and arises from the surface of the M. omohyoideus and M. sternohyoideus-complex, running in direction towards the M. scapulo-humeralis posterior, where it inserts inside connective tissue situated between the latter and the M. serratus anterior superficialis. A reliable separation of the two units into M. subcoracoideus und M. subscapularis, respectively, is not possible due to the non-skeletal origin and insertion areas in *Trogonophis wiegmanni*. Therefore, both parts are here treated as M. subcoraco-scapularis.

The **M. sternocoracoideus** arises from the sternum and runs along and inserts into the scapulocoracoid, arching slightly anterior. Especially in *Trogonophis wiegmanni* it represents the linkage between sternum and scapulocoracoids as described by Gans [55]. In *Trogonophis wiegmanni* the M. sternocoracoideus is a relatively small muscle that is strongly associated with the M. omohyoideus und M. sternohyoideus-complex, by which it is also covered distally.

##### Amphisbaenidae (*Cynisca leucura*)

**Bones of the shoulder girdle**

**Fig. 9:**
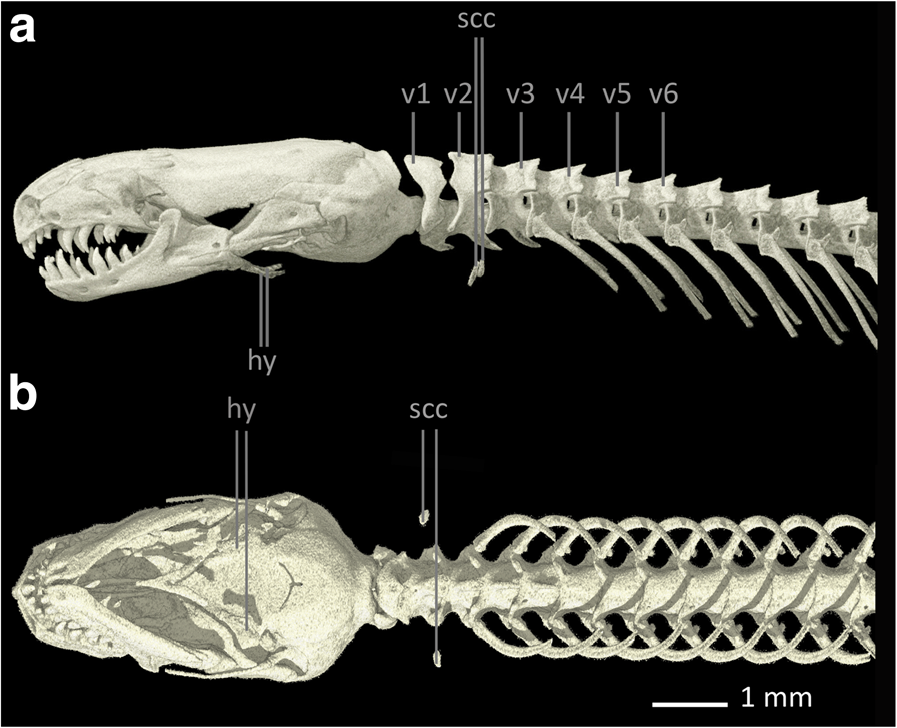
Skull, anterior part of the vertebral column and pectoral region of *Cynisca leucura*. **a** lateral view, **b** ventral view. Abbreviations: hy: hyoids, scc: scapulocoracoids, v: vertebra

Kearney [8] and Kearney & Stuart [32] state that *Cynisca* possesses no shoulder girdle elements, but Zangerl [30] already assumed that vestiges of the sternum and other shoulder girdle elements are probably present in all amphisbaenians. Here we can confirm the presence of rudimentary shoulder girdle elements in *Cynisca leucura* as small, rod-like, bony structures that we interpret as **scapulocoracoids**, which are located at the level of the third vertebra (see Fig. 9).

**Muscles of the shoulder girdle**

**Overview of present muscles**

**Fig. 10:**
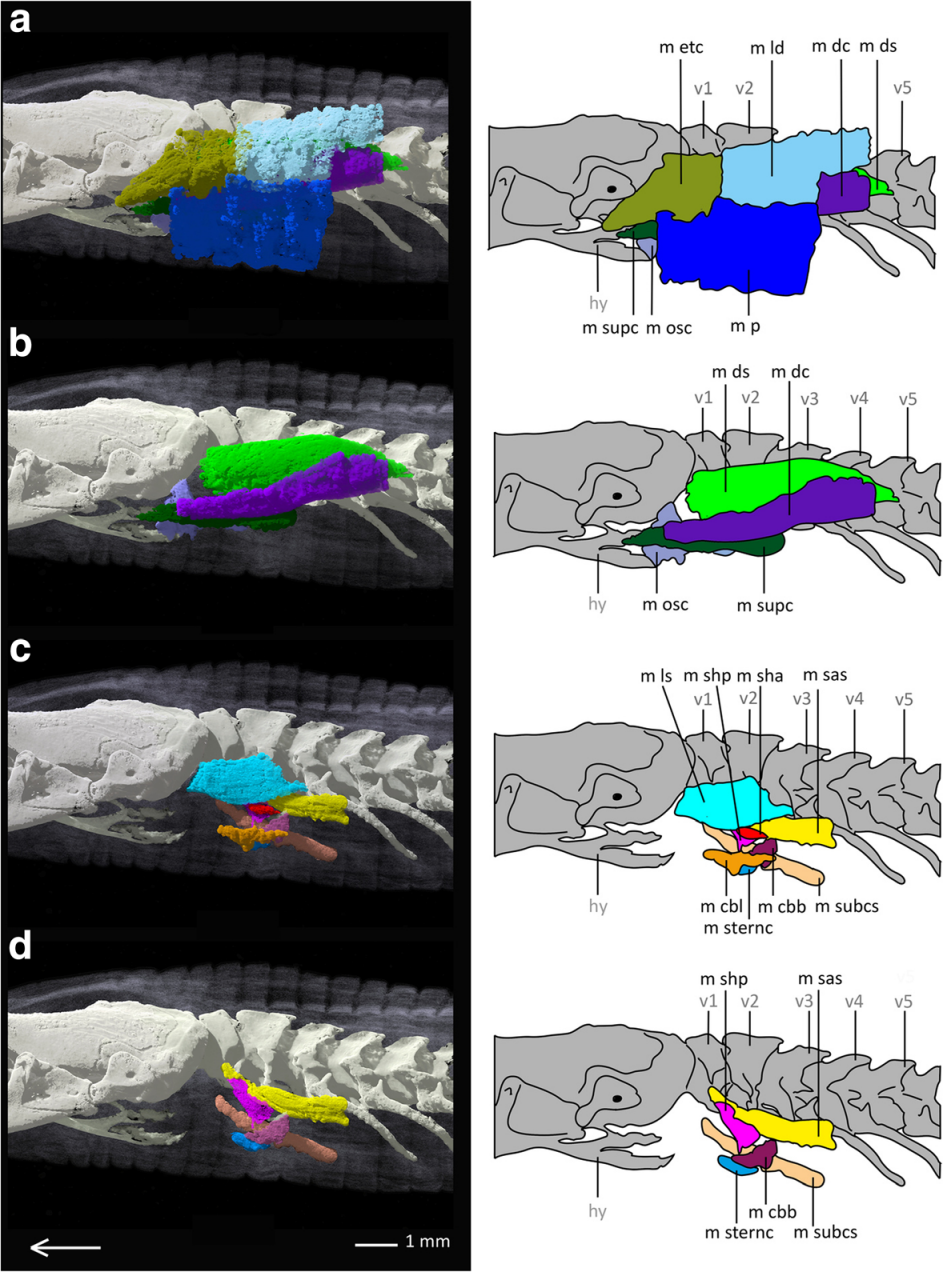
Pectoral muscles of *Cynisca leucura* in lateral view. **a** distal superficial pectoral muscles of *Cynisca leucura*, **b** medial superficial pectoral muscles of *Cynisca leucura,* **c** distal subjacent pectoral muscles of *Cynisca leucura*, **d** medial subjacent pectoral muscles of *Cynisca leucura*. Array shows anterior direction, abbreviations: hy: hyoid, m cbb: M. coraco-brachialis brevis, m cbl: M. coraco-brachialis longus, m dc: M. deltoideus clavicularis, m ds: M. deltoideus scapularis, m etc: M. episternocleidomastoideus and M. trapezius-complex, m ld: M. latissimus dorsi, m ls: M. levator scapulae, m osc: M. omohyoideus and M. sternohyoideus-complex, m p: M. pectoralis, m sas: M. serratus anterior superficialis, m sha: M. scapulo-humeralis anterior, m shp: M. scapulo-humeralis posterior, m sternc: M. sternocoracoideus, m subcs: M. subcoraco-scapularis, m supc: M. supracoracoideus, v: vertebra

The forelimbs of *Cynisca leucura* are absent and so are the muscles belonging to the upper arm, i.e. M. biceps brachii, M. brachialis, and M. triceps. Apart from these all other 17 muscles of the shoulder girdle are present (see Fig. 10a, b for superficial and Fig. 10c, d for subjacent muscles), whereas M. omohyoideus and M. sternohyoideus, as well as M. episternocleidomastoideus and M. trapezius again form complexes.

**Superficial muscle layers**

The **M. latissimus dorsi** of *Cynisca leucura* is a large, flat, superficial muscle that runs dorsolaterally, similar to the other investigated taxa. However, it is smaller than, e.g., in *Trogonophis wiegmanni*. The muscle’s orientation is very similar to that of *Meroles cuneirostris* and *Bipes biporus*. It arises dorsally from a layer of connective tissue near the skin and runs ventrally, whereas it ends further ventrally in the anterior part than in the posterior part. It inserts into a ventral layer of connective tissue near the skin and into the surface of the M. deltoideus clavicularis. The M. latissimus dorsi overlies the M. deltoideus scapularis, the M. deltoideus clavicularis and the M. supracoracoideus from the dorsal site.

The **M. episternocleidomastoideus and M. trapezius** form a complex in *Cynisca leucura*, which is anteriorly connected to the M. latissimus dorsi. As a result, it is difficult to differentiate between the M. episternocleidomastoideus and M. trapezius complex and the M. latissimus dorsi, because some of their fibres are fused. The M. episternocleidomastoideus and M. trapezius-complex arises near the vertebral column and runs with parallel fibres in a cranioventral direction, inserting near the cranium. As in *Trogonophis wiegmanni, Bipes biporus* and *Blanus strauchi* it is the most anteriorly positioned muscle mass. The muscle is slightly smaller than in *Blanus strauchi*, and in general very similar to the M. episternocleidomastoideus and M. trapezius-complex of *Trogonophis wiegmanni.*

**M. omohyoideus and M. sternohyoideus** form a trapezoid-shaped complex, which arises from the surface of the M. coraco-brachialis longus by a ventral branch in direction of the scapulocoracoid, extending anteriorly. It inserts into the hyoid and into an area further dorsally, near the cranium. The dorsal part of the complex arises from the surface of the M. supracoracoideus. In its dorsal half the complex is overlain for two thirds by the M. episternocleidomastoideus and M. trapezius-complex.

The **M. deltoideus scapularis** appears as a large, massive muscle, which is completely covered by the M. latissimus dorsi. It shows a more strand-like morphology than in *Blanus strauchi* and is comparable in its structure to *Trogonophis wiegmanni*. The muscle arises from the surface of the M. levator scapulae as well as from a large part of the surface of the M. longissimus, similar to *Trogonophis wiegmanni*. It runs with parallel fibres along the ventral side of the body and inserts near the cranium into an area of connective tissue.

Ventrally to the M. deltoideus scapularis the **M. deltoideus clavicularis** is located. It follows the course of the M. deltoideus scapularis and covers the latter’s lower third. It arises from a large part of the surface of the M. deltoideus scapularis and runs cranially, until it inserts into the surface of the M. supracoracoideus, which is located ventrally to it. The muscle closely approaches the scapulocoracoid, but there is no contact. In its posterior region the muscle is more strongly associated with the M. deltoideus scapularis than in, e.g., *Blanus strauchi*.

The **M. supracoracoideus** arises fibrously from the skin, i.e. from connective tissue located beneath the skin in the lateral area of the body, at the level of the scapulocoracoids. It runs anteriorly and inserts near the cranium, but slightly more anteriorly than the M. deltoideus clavicularis. It is very similar in its morphology to that of *Trogonophis wiegmanni*, but it is much larger and slightly overlies the M. deltoideus scapularis dorsally.

The **M. pectoralis** completes the muscles of the shoulder girdle ventrally, like in all other examined taxa. It runs in several thin, partly overlapping layers across the scapulocoracoids anteriorly, where it contacts the M. episternocleidomastoideus and M. trapezius-complex anteriorly and the M. latissimus dorsi posteriorly. It reaches more dorsally than in all other examined species*.* It arises from a ventral layer of connective tissue and inserts anteriorly into another layer of connective tissue, which is located more dorsally and into the surface of the M. episternocleidomastoideus and M. trapezius complex. Posteriorly it inserts into the surface of the M. latissimus dorsi.

**Subjacent muscle layers**

The **M. serratus** **anterior superficialis** is located proximally to the M. deltoideus scapularis, by which it is covered in the anterior area. In its morphology it is similar to that of *Blanus strauchi*. It arises posteroproximally from the origin of the M. levator scapulae from the first vertebra, and runs posteroproximally along the ventral side of the specimen, slightly touching the scapulocoracoid. It is associated with the M. scapulo-humeralis posterior in its anterior region and inserts into a layer of inter-muscular connective tissue.

The **M. scapulo-humeralis anterior** is a small, rotund muscle that is located between the other shoulder girdle muscles. Proximally it is surrounded by the M. scapulo-humeralis posterior, the M. levator scapulae and the M. subcoraco-scapularis, and distally by the M. deltoideus clavicularis and M. supracoracoideus. It arises from the scapulocoracoid and runs anteriorly, similar to *Trogonophis wiegmanni*, and inserts into the M. scapulo-humeralis posterior.

The **M. scapulo-humeralis posterior** is an elongated, flat muscle, which arises from the scapulocoracoid and runs anteroproximally into the torso of the specimen, like in *Trogonophis wiegmanni*. It inserts into the M. serratus anterior superficialis and is covered by the distally located M. deltoideus scapularis, M. deltoideus clavicularis, and M. supracoracoideus.

The **M. levator scapulae** is located proximally to the M. deltoideus scapularis, by which it is completely covered, only anteriorly it runs beyond the latter’s extent. It arises from the cranium and runs posteriorly, until it inserts into the M. longissimus.

Proximally to the M. supracoracoideus and M. coraco-brachialis longus the **M. coraco-brachialis brevis** is located, which shows a crescentic shape and arises from the scapulocoracoid. It runs anteriorly and inserts into the M. sternocoracoideus.

Distally to the M. coraco-brachialis brevis, the **M. coraco-brachialis longus** is located, which arises from the surfaces of the M. supracoracoideus and M. coraco-brachialis brevis, running anteriorly. In its shape it is quite similar to *Meroles cuneirostris* and *Blanus strauchi*, but its position is different. The muscle inserts into the surface of the M. omohyoideus and sternohyoideus-complex, as well as into the surface of the M. supracoracoideus, whose course it follows over a certain distance. It is overlain by the M. pectoralis distally.

The **M. subcoraco-scapularis** arises from the ventral area, i.e. from a layer of connective tissue inside the body. From there it runs in direction towards the scapulocoracoid, into which it inserts, but it also runs further anteriorly and inserts into the surface of the M. levator scapulae. In *Cynisca leucura* this muscle is markedly larger and more elongated than in all other examined species.

Ventrally to the M. subcoraco-scapularis the **M. sternocoracoideus** is situated, which represents a relatively small, rotund muscle. It arises from a layer of connective tissue near the M. omohyoideus and M. sternohyoideus complex and runs posteriorly in direction of the scapulocoracoid to the M. coraco-brachialis brevis, inserting into the latter’s surface. Distally it is overlain by the M. coraco-brachialis longus and by the M. pectoralis.

See Additional file 5: Table S5 “pectoral muscle origins and insertions” for an overview of all muscle origins and insertions within the different amphisbaenian taxa.

#### Rhineurid amphisbaenians

##### Rhineuridae (*Rhineura floridana*)

**Bones of the shoulder girdle**

**Fig. 11:**
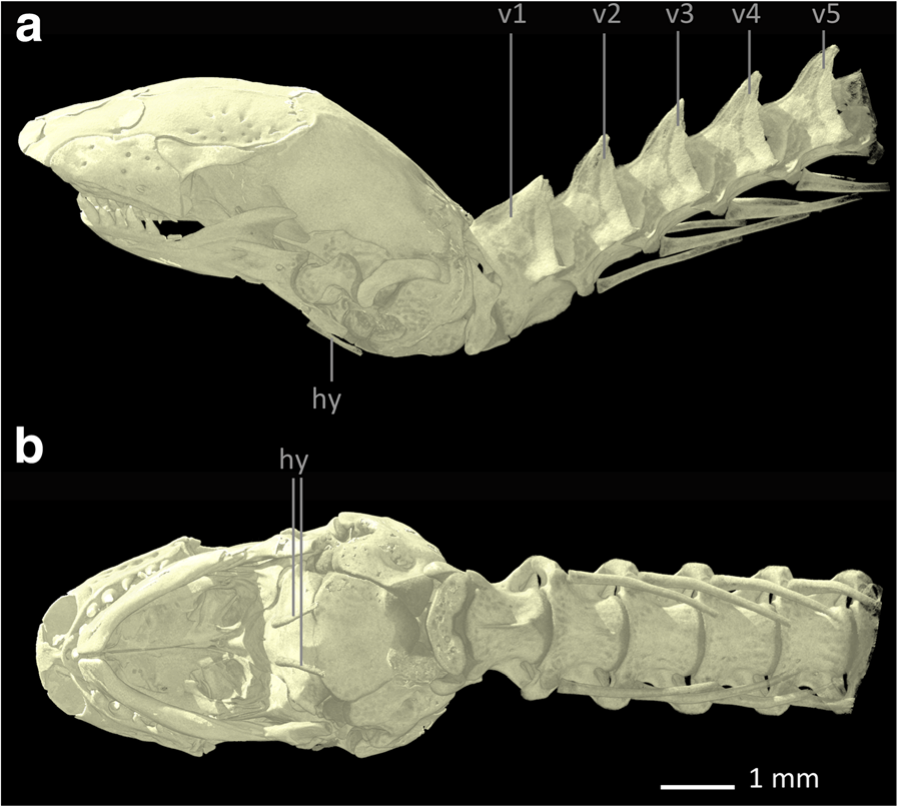
Skull, anterior part of the vertebral column and pectoral region of *Rhineura floridana*. **a** lateral view, **b** ventral view. Abbreviations: hy: hyoids, v: vertebra

In *Rhineura floridana* no elements of the pectoral girdle and the forelimbs are present [8]. This condition was confirmed in our μ-CT-scans of *Rhineura floridana* (see Fig. 11).

**Muscles of the shoulder girdle**

The muscles in the ancestral shoulder girdle region of *Rhineura floridana* differ strongly from the shoulder girdle muscles of all other examined amphisbaenian species, and consist mostly of elongated, longitudinal muscles. We found 18 muscles in this area, which consist of lateral and pectoral strands and are morphologically similar to the remaining longitudinal muscles in the anterior trunk region. For this reason, none of the muscles can be homologized with the shoulder muscles of the other examined taxa, and we therefore decided to assign numbers, rather than names, to each muscle. All muscles of the ancestral shoulder girdle region of *Rhineura floridana* are single muscles, although there are some strong associations between the 4th and 5th lateral and between the 1st, 2nd, 3rd and 4th pectoral muscle strands. There are no muscle complexes.

**Superficial muscle layers of the considered shoulder region**

**Fig. 12:**
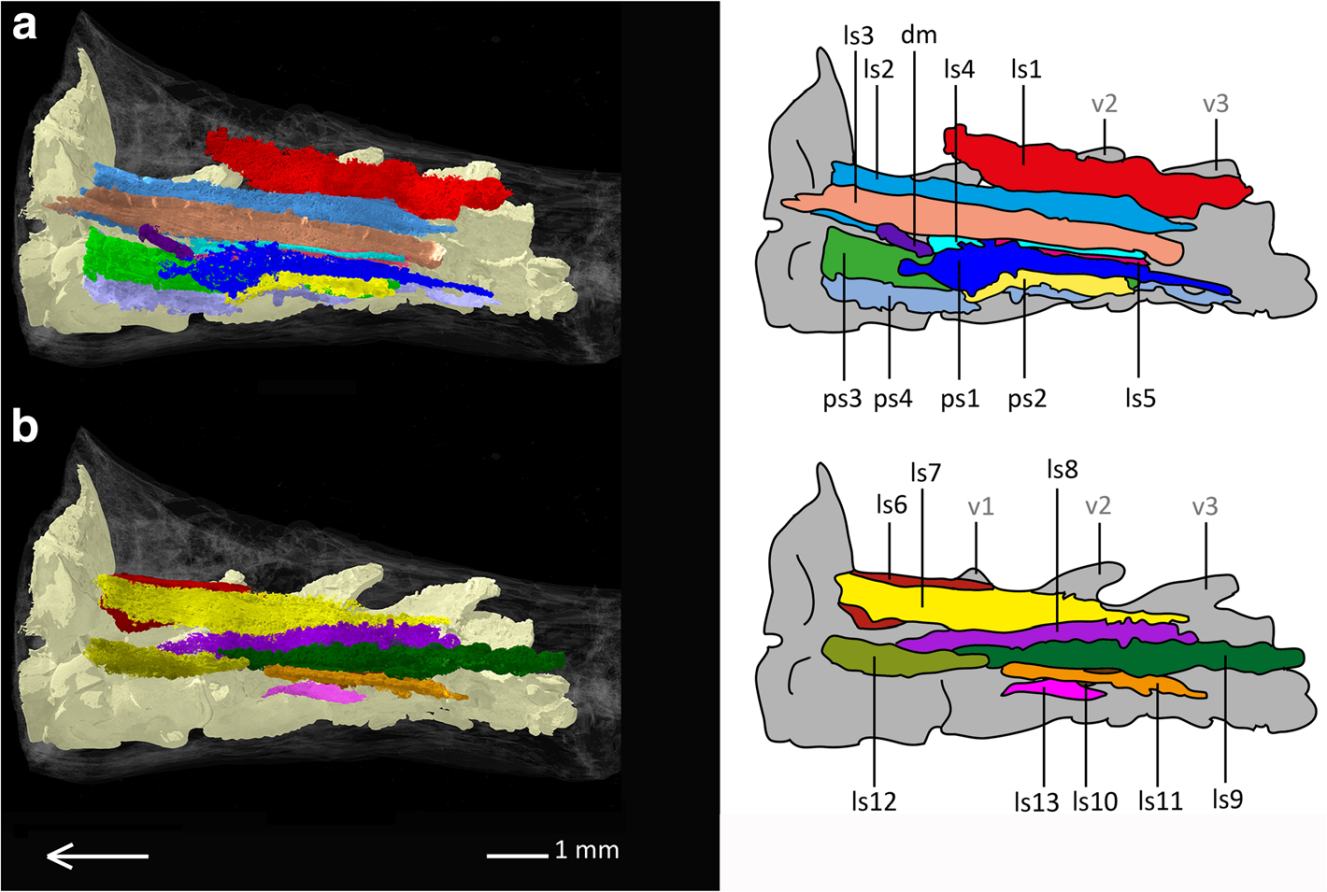
Pectoral muscles of *Rhineura floridana* in lateral view. **a** superficial pectoral muscles of *Rhineura floridana*, **b** subjacent pectoral muscles of *Rhineura floridana*. Array shows anterior direction, abbreviations: dm: discrete muscle, ls: lateral strand, ps: pectoral strand, v: vertebra

Within the ancestral shoulder region of *Rhineura floridana*, ten superficial muscles (see Fig. 12a) are present that can be divided into five lateral strands (1st- 5th, **ls**), four pectoral strands (1st- 4th, **ps**) and one discrete muscle (**dm**).

**The 1st lateral muscle** runs as a thick and large muscle from the spinal process of the third vertebra, ventrally to the 1st dorsal strand, and inserts near the anterior end of the 3rd neck muscle inside a narrow area of connective tissue in the postcranial region directly behind the skull.

**The 2nd lateral muscle** is a thin layered muscle that arises from both the skin and an aponeurosis at the level of the third vertebra, running anteriorly to the lower postcranial region behind the skull, where it inserts into the cranium through slender fibres. It partly covers the course of the 1st lateral muscle. It is surrounded by the 1st lateral muscle and the 7th lateral muscle dorsally as well as by the 3rd lateral muscle ventrally.

**The 3rd lateral muscle** runs ventrally to the 2nd lateral muscle, which it also partially covers. It arises ventrally to the origin of the 2nd lateral muscle from both the skin and an aponeurosis right beneath the skin, running anteriorly to the same lower postcranial region as the 2nd lateral muscle, where it inserts into an area of connective tissue near the skin. The 3rd lateral muscle shows mostly the same shape as the 2nd lateral muscle, including the overall elongated and thin-layered condition.

**The 4th lateral muscle** is very thin and flat. It arises from an area of connective tissue near the skin and runs anteriorly to insert inside an inter-muscular region at the level anterior to the transverse process of the first vertebra. The muscle becomes slightly broader anteriorly. It is surrounded by the 3rd lateral strand dorsally and the 1st pectoral strand ventrally.

**The 5th lateral muscle** is very similar to the 4th and strongly associated with it. It has the same origin and follows its course proximally to insert partly into the same inter-muscular region as the 4th lateral muscle as well as inside the surface of the 4th lateral muscle. It is thin and very flat. Distally it is partly overlain by the 1st pectoral strand.

**The 1st pectoral strand** runs ventrally to the 4th and 5th lateral strands and arises at the posterior level of the third vertebra from a tiny inter-muscular region. It is fibrous and thin and runs along the transverse process of the third to first vertebrae along the vertebral column. It becomes flatter and broader anteriorly, until it inserts inside an inter-muscular region at the level of the transverse process of the first vertebra.

**The 2nd pectoral strand** runs ventrally to the 1st pectoral strand, with which it is strongly associated. At some points, the 1st and 2nd pectoral might be even seen as a single muscle. The 2nd pectoral muscle arises from second rib at the level of the third vertebra and runs anteriorly to insert into the skin at the level of the transverse process of the first vertebra. Like the 1st pectoral strand, it is overall very flat.

**The 3rd pectoral strand** is a bulky muscle that runs proximal to the 1st and 2nd pectoral strand, by which it is also partly overlain. It arises from the second rib at the level of the third vertebra and runs anteriorly to insert into an inter-muscular region near the lateral postcranial region right behind the skull. It partly encases the 4th pectoral muscle which is orientated proximally.

**The 4th pectoral strand** runs proximally and ventrally to the 3rd pectoral strand. It arises ventrally from the transverse process of the third vertebra and runs anteriorly to insert near the postcranial region into the transverse process of the 1st vertebra as well as into an inter-muscular region near the lateral postcranial region. It consists of a flat and thin muscle layer that tapers along the transverse processes from the third to the first vertebra. It is the most ventrally positioned muscle.

The only **discrete muscle** in *Rhineura floridana* is positioned relatively far anterior within the animal. It arises from a tiny inter-muscular region near the skin at the level of the first vertebra. It is a small, elongated muscle that runs anteriorly and inserts into inter-muscular region near the skin. It has no connection to the other muscle strands and shows a dorsally trending course towards the cranium. It is smaller than all other muscles described above.

**Subjacent muscle layers of the considered shoulder girdle region**

Within the ancestral shoulder girdle region of *Rhineura floridana* there are eight subjacent muscle strands (see Fig. 12b), consisting of the eight lateral strands (6th to 13th, **ls**).

**The 6th lateral strand** is a short, but massive muscle. It arises from an inter-muscular region near the lateral side of the anterior end of the neural spine of the second vertebra and runs anteriorly, passing the neural arch of the first vertebra laterally and inserting into the lower cranium.

**The 7th lateral strand** is more elongated than the 6th. It arises from a rounded elevation on the neural arch of the third vertebra and runs anteriorly along the vertebral column, covering the third to first vertebra. It touches the 6th lateral strand near the latter’s point of origin and inserts into the lower cranium distally to the insertion of the 6th lateral strand.

**The 8th lateral strand** is not as large as the 7th lateral strand. It originates from the transverse process of the third vertebra and runs anteriorly along the transverse processes of the second and first vertebrae, inserting into an inter-muscular region anteriorly to the transverse process of the first vertebra. It is distally overlain by the 2nd and 3rd lateral strand.

**The 9th lateral strand** runs ventrally to the 8th lateral strand, to which it is also very similar. It tapers along the transverse processes of the vertebral column anteriorly to the transverse process of the first vertebra, where it inserts into the 8th lateral strand slightly posterior to the latter’s insertion side. The 8th and 9th lateral strands are partly associated. The 9th lateral strand is also distally overlain by the 2nd and 3rd lateral strand.

**The 10th lateral strand** is a very small and short muscle. It arises from the beginning of the second rib near the vertebral column at the level of the third vertebra and runs anteriorly to insert into the beginning of the first rib near the vertebral column. Due to its position, the 10th lateral strand can be seen as an inter-rib muscle, because it links the second and first rib with each other.

**The 11th lateral strand** is an elongated, slender muscle strand and larger than the 10th lateral strand. It arises from the distal tip of the second rib at the level of the third vertebra and runs anteroproximally to the 5th lateral strand, inserting into an inter-muscular region near the insertion of the first rib at the level of the second vertebra.

**The 12th lateral strand** is situated anteriorly to the 11th lateral strand and is of largely similar size. It arises from an inter-muscular region near the 3rd lateral strand and runs anteriorly towards the cranium to insert into the first vertebra laterally.

**The 13th lateral strand** arises from the head of the second rib and runs anteriorly to the head of the first rib, where it also inserts. It is a small muscle similar to the 10th lateral strand, but it runs more ventrally, on the ventral side of the ribs. It might be viewed as an inter-rib muscle, because it creates a linkage between the first and second rib. Distally it is completely covered by the 2nd pectoral strand.

## Discussion

### Limb reduction, muscles and function

Except *Rhineura floridana* that features 18 muscles within the pectoral region, all amphisbaenian taxa examined in this study possess the ancestral number of 17 shoulder and pectoral muscles. Some of these muscles occur as complexes, such as the M. omo- and sternohyoideus- complex and the M. episternocleidomastoideus and trapezius complex. In some cases the muscles are also strongly associated with the surrounding neck- and dorsal musculature, with which they seem to form a functional unit. This applies especially to the M. episternocleidomastoideus and trapezius-complex, the M. omo- and sternohyoideus-complex, the M. deltoideus scapularis, and to the M. levator scapulae. In all examined amphisbaenians as well as in *Meroles cuneirostris*, the M. latissimus dorsi, the M. episternocleidomastoideus and the M. trapezius-complex form an outer muscle arrangement dorsally, whereas the M. pectoralis covers the pectoral region ventrally. In Blanidae, Trogonophidae and Amphisbaenidae, the dorsally covering muscles are elongated ventrally and approach the dorsally elongated M. pectoralis laterally. In *Blanus* there is no uninterrupted outer muscle cover, whereas in *Trogonophis* and *Cynisca* there is an continuous cover in form of a thin external muscle layer, which surrounds the entire pectoral area right beneath the skin. A notable feature observed in all amphisbaenian taxa is that the shoulder girdle agglomerate is shortened and shifted anteriorly relative to the ancestral position as seen in *Meroles cuneirostris* (see Additional file 2: Text S2, Additional file 3: Figure S3 and Additional file 4: Figure S4). In most squamates, the pectoral girdle and humeral insertion are typically positioned at the level of the 5th vertebra or further posteriorly [29]. In our CT-scans, the pectoral girdle of *Meroles cuneirostris* occurs at the level of the 7th vertebra, whereas in *Bipes* it is found at the level of the 2nd vertebra which in external view makes the limbs appear to be positioned directly behind the head [8, 29]. This anterior shift of pectoral elements is also visible in the shoulder muscles. In *Meroles cuneirostris* the superficial layer of the shoulder girdle muscles reaches from the skull to the 11th vertebra and the subjacent muscles cover the area between the skull and the 7th vertebra, whereas in amphisbaenians, the superficial layer reaches from the skull to the 5th vertebra in most taxa and the subjacent layer covers the region between the skull and the 3rd to 4th vertebra (see Results for details).

In tetrapods, the vertebral column is generally regionalized into presacral (cervical, thoracic), sacral and caudal series based on changes in skeletal morphology and integration with the appendicular skeleton. Both the number of regions, their morphologies, and the number of elements composing them are variable across taxa [58, 59]. In Amphisbaenia, the positions of discrete anatomical boundaries that differentiate the cervical and thoracic regions of the vertebral column in other squamate taxa are spatially dissociated: Unfused intercentra are restricted to the atlas-axis complex and ribless vertebrae are limited to the first 1–2 elements posterior to the atlas-axis, whereas ventral hypapophyses variably occur posteriorly to the tenth precloacal vertebra, generally consistent with the recognition of morphometric vertebral shape boundaries in model taxa [30, 60, 61].

The relationship of the pectoral girdle to the axial skeleton in amphisbaenians is similarly spatially offset, suggesting dissociation between developmental domains. Dorsal vertebrate and ribs are patterned from paraxial mesoderm by collinear *Hox* gene expressioni, whereas the appendicular skeleton, including the sternum, ventral ribs, girdles, limbs, and most associated muscles, are patterned from lateral plate mesoderm by distinct *Hox* expression [62]. These two patterning histories are recognized as distinct primaxial and abaxial domains, respectively [63–66] with the embryonic boundary between their tissues recognized as the lateral somatic frontier [64, 65]. Head and Polly (2015) [61] suggested that the regional boundaries in the pre-cloacal primaxial domain of elongate, limb-reduced lizards, including amphisbaenians, is not deregionalized compared to limbed taxa, so the number of cervical vertebrae is not reduced in Amphisbaenia. This finding is also supported by the presence of intercentral remants on the centra of the anterior vertebrae 1–5 in our investigated taxa, showing that both amphisbaenians and lacertids share a rather similar pattern in their cervical region.

Evolutionary dissociation, such as developmental repatterning of one domain relative to another, is a potential mechanism for spatial reorganization of tissues [67]. In the case of amphisbaenians, displacement of the pectoral girdle anterior to the ribcage and other cervico-thoracic anatomical markers relative to limbed squamates may represent a modular shift along the lateral somatic frontier. Loss of ventral ribs that link the sternum with the dorsal ribcage in other taxa, may have enabled a successful spatial shift in the relationship of the girdle to the rest of the skeleton. Within all amphisbaenian families except *Bipes* a restructuring of the shoulder muscles can be observed, which is likely due to the loss of humerus and forelimbs. In the original condition of a quadrupedal tetrapod, the M. deltoideus scapularis, the M. deltoideus clavicularis, the M. latissimus dorsi, the M. supracoracoideus and the M. pectoralis are closely associated with the deltopectoral crest of the humerus and play an important role in locomotion [46, 52, 57, 68, 69]. In all amphisbaenians except *Bipes*, the attachment sites of the respective muscles have changed relative to the ancestral condition due to the reduction of the humerus. It is likely, therefore, that the function of the single shoulder muscles in *Blanus*, *Trogonophis*, *Cynisca* and *Rhineura* is no longer the ancestral, humerus-associated function. The muscles that originally served as retractors and protractors of the humerus [70] are very closely associated within these families, forming a coherent muscle mass that spans the whole pectoral region. We hypothesize that muscles that were ancestrally important for the function of the limbs are those which are the most restructured within Amphisbaenia.

Although the functional role of the different shoulder muscles is still unknown for Amphisbaenia, we also predict that the evolutionary rearrangement of the muscles was not primarily driven by developmental constraints but by functional demands. This view is supported by the observation that there is a clearly visible division into two muscle layers, a superficial and a subjacent layer, which appear as clearly separated, ring-like structures in axial view. Both layers contain the same muscles across the different species (whereas *Rhineura* is a special case and may require additional investigations), but the muscles within each layer have different developmental origins and can be considered either axial shoulder or intrinsic limb muscles [71, 72]. If developmental factors were primarily guiding muscle rearrangement, such a division would not be expected.

### Alternative origins and insertions of muscles

Our study shows that muscle reduction cannot be reliably predicted from bone anatomy alone [6]. Instead, origin and insertion sites of the shoulder muscles shift to non-skeletal areas, such as surrounding muscles, skin or connective tissue. Tsuihiji et al. [73] mention the same phenomenon for snakes, which have entirely reduced their bony shoulder girdle [74–76]. In the latter, the M. episternocleidomastoideus originates from non-skeletal elements like the surface of other muscles, i.e. the cutaneous muscle layer composed of the lamina muscularis secunda and the M. squamosquamales underlying the skin in typhlopids, the medial aspects of the M. costocutaneous superior and inferior and the M. squamosquamales in *Cylindrophis rufus*, or the M. costomandibularis in *Acrochordus granulatus*, whereas the muscle origin itself remains in the same region where the shoulder girdle was ancestrally located [73]. Given there is no additional information on snake pectoral muscle anatomy, a further comparison with amphisbaenians is not possible. Taken together, however, these findings suggest that muscle origin sites are more closely connected to bone topology than to bone identity [77]. Muscles do not seem to be dependent on their original attachment sites, but may stay conserved even after the reduction of bones, and bone reduction does not necessarily result in the loss of associated muscles. This view of a decoupling of skeletal and connective tissues is also supported by developmental evidence, indicating a high degree of developmental autonomy within the musculoskeletal system [22, 78].

### Synapomorphies and autapomorphies

**Fig. 13:**
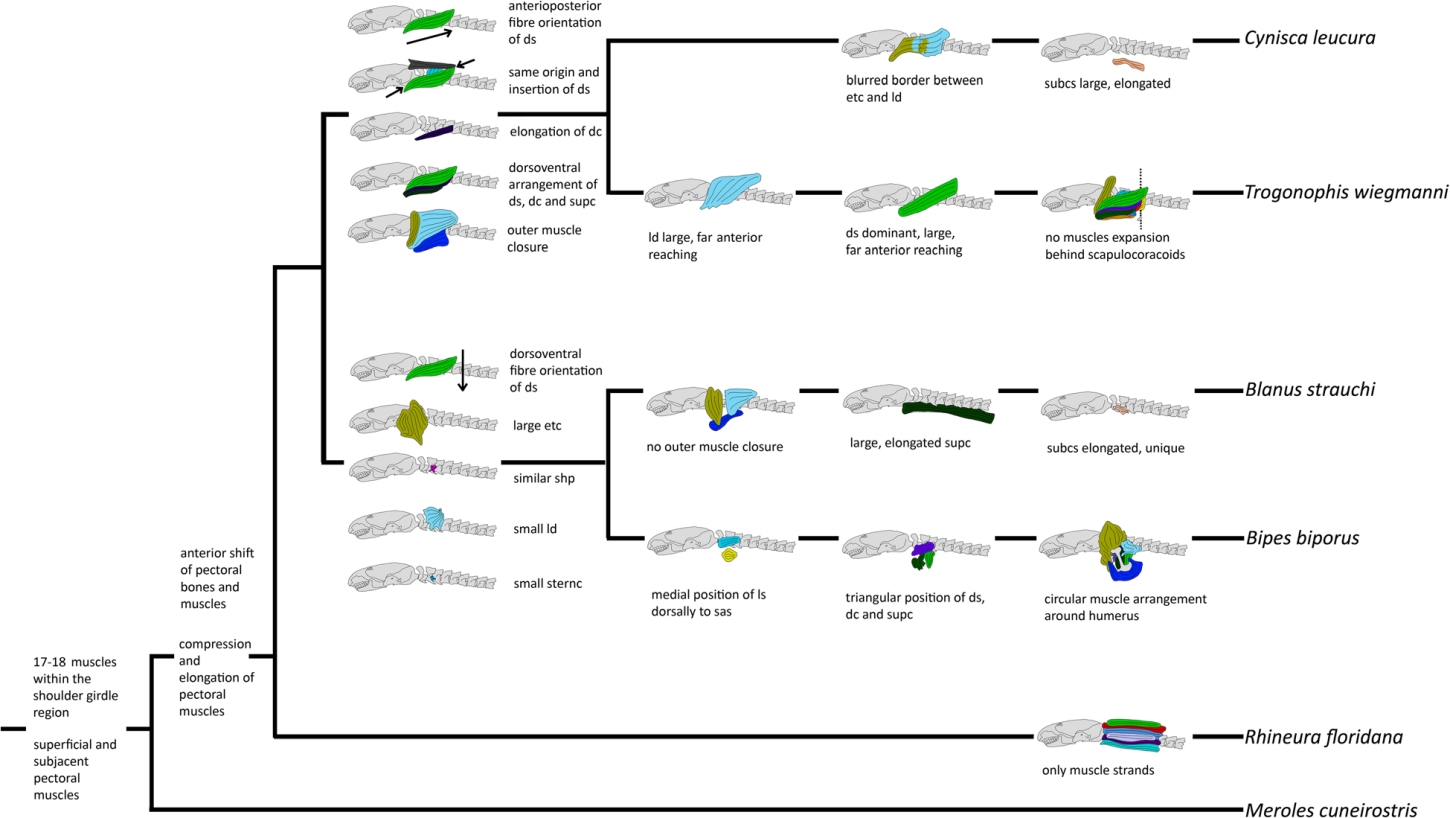
Amphisbaenian phylogeny based on Vidal et al. (2008) [35] and Hipsley & Müller (2014) [41]. Schematic pictures and text boxes show synapomormies and autapomorphies concerning pectoral muscles, abbreviations: dc: M. deltoideus clavicularis, ds: M. deltoideus scapularis, etc: M. episternocleidomastoideus and M. trapezius-complex, ld: M. latissimus dorsi, ls: M. levator scapulae, sas: M. serratus anterior superficialis, shp: M. scapulo-humeralis posterior, sternc: M. sternocoracoideus, subcs: M. subcoraco-scapularis, supc: M. supracoracoideus

**Fig. 14:**
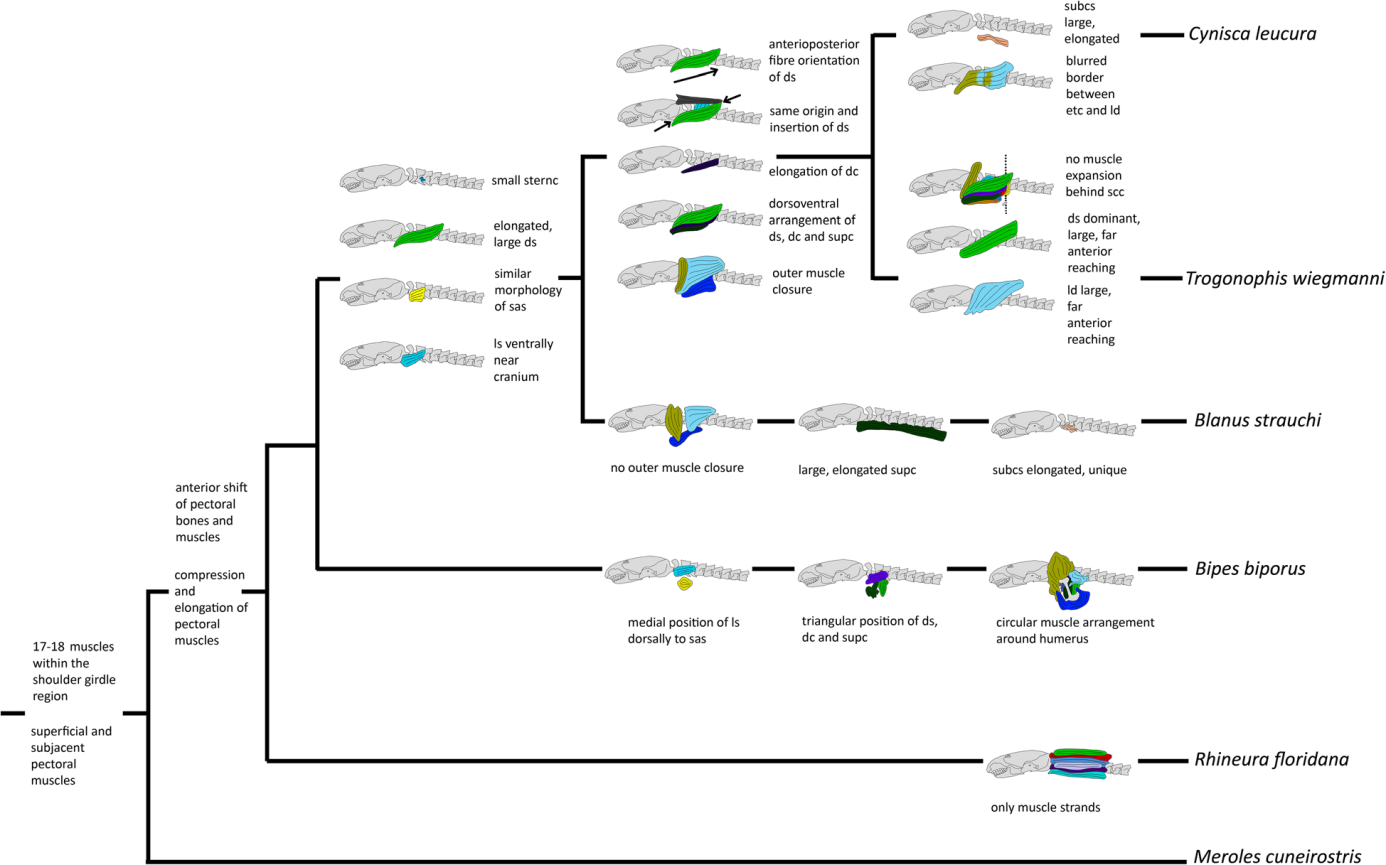
Amphisbaenian phylogeny based on Pyron et al. (2013) [36]. Schematic pictures and text boxes show synapomormies and autapomorphies concerning pectoral muscles, abbreviations: dc: M. deltoideus clavicularis, ds: M. deltoideus scapularis, etc: M. episternocleidomastoideus and M. trapezius-complex, ld: M. latissimus dorsi, ls: M. levator scapulae, sas: M. serratus anterior superficialis, sternc: M. sternocoracoideus, subcs: M. subcoraco-scapularis, supc: M. supracoracoideus, scc: scapulocoracoids

Currently there are two, somewhat conflicting hypotheses on amphisbaenian phylogeny, differing primarily in the position of *Bipes* and *Blanus*. The phylogeny by Vidal et al. [35] and Hipsley & Müller [41] (see Fig. 13) suggests a sister group relationship between *Blanus* and *Bipes* relative to Trogonophidae and Amphisbaenidae, whereas in the phylogeny by Pyron [36] (see Fig. 14) *Blanus* is positioned as a sister taxon to the terminal sister taxa Trogonophidae and Amphisbaenidae. In both phylogenies, Rhineuridae has a basal position and is a sister taxon to all other amphisbaenians. Longrich et al. [77] show an unresolved relationship for the respective taxa.

All examined species, including *Meroles cuneirostris,* share the same condition of having 17–18 muscles within the pectoral area, divided into a superficial and a subjacent layer. Two synapomorphies that all amphisbaenians share are an anterior shift of pectoral bones and muscles and a compression and elongation of pectoral muscles relative to the ancestral position seen in other squamates, e.g. *Meroles cuneirostris* (see Figs. 13 and 14).

Under the assumption that *Bipes* and *Blanus* were sister taxa, there would be five synapomorphies supporting this grouping. These are the dorsoventral muscle fibre orientation of the M. deltoideus scapularis, a large M. episternocleidomastoideus and M. trapezius-complex, a similar morphology of the M. scapulo-humeralis posterior, a relatively small M. latissimus dorsi and M. sternocoracoideus (see Fig. 13).

Alternatively, if *Blanus* was sister to Trogonophidae and Amphisbaenidae, then this would be supported by four synapomorphies; a relatively small M. sternocoracoideus, an elongated and large M. deltoideus scapularis, a similar morphology of the M. serratus anterior superficialis and a ventral position of the M. levator scapulae near the cranium (see Fig. 14).

The currently undisputed sister-group relationship between *Trogonophis*/Trogonophidae and *Cynisca*/Amphisbaenidae is supported by five synapomorphies. These are the anteroposterior muscle fibre orientation of the M. deltoideus scapularis, and the same origin and insertion of the M. deltoideus scapularis, which originates from the M. levator scapulae and the M. longissimus and runs with an identical course in both taxa to the insertion point in an area of connective tissue near the cranium. Furthermore, *Trogonophis* and *Cynisca* both show an elongation of the M. deltoideus clavicularis; a dorsoventral arrangement of the M. deltoideus scapularis, the M. deltoideus clavicularis and the M. supracoracoideus; and an outer muscle closure consisting of the M. latissimus dorsi and the M. episternocleidomastoideus and M. trapezius-complex from the dorsal side and M. pectoralis from the ventral side, with a close association between these muscles (see Figs. 13 and 14).

Rhineuridae is the only amphisbaenian taxon in which all 17 shoulder girdle muscles show a strand-like morphology, except for one discrete muscle. Altogether, *Rhineura* possesses 18 muscles within the shoulder girdle region. One may hypothesize that the 17 strand-shaped muscles are homologous to the 17 shoulder muscles of other amphisbaenians concerning their topology and position within the pectoral region, and because of the sister-group relationship between *Rhineura* and all other amphisbaenians [36, 41], but this statement must be rendered speculative until further evidence is given. Furthermore, such a hypothesis would imply an evolutionary restructuring from true shoulder muscles into muscle strands in *Rhineura*, including a separate evolutionary history for the 18th muscle.

The results of our study also represent the first, at least indirect, morphological support of the molecular-based hypothesis that shovel-headed amphisbaenians evolved independently, i.e. shovel-headed taxa other than *Rhineura,* i.e. *Aulura* and *Leposternon* from South America and *Dalophia* and *Monopeltis* from Africa, belong to Amphisbaenidae and are thus not monophyletic with the former [32]. Shovel-headed forms show a facial area that is deeply flattened into an oval, spatulate shape [29]. If shovel-headed forms were monophyletic, they all would have to show a strand-like pectoral morphology similar to *Rhineura*, or alternatively, *Rhineura* would have to share the apomorphic characters seen in Amphisbaenidae/Trogonophidae. Whereas we admit that the only examined amphisbaenid taxon in our study was *Cynisca leucura*, which has a rounded head, the sister-group relationship between *Cynisca* and *Trogonophis,* with both showing a very similar, derived pectoral muscle morphology (see above), implies that also other representatives of Amphisbaenidae, including the shovel-headed forms, will show a similar pattern that is different from that of *Rhineura.* Although further studies (and more taxon sampling) are required, we thus consider our observations sufficient support for the hypothesis that shovel-headed morphologies evolved convergently and independently within Amphisbaenidae and Rhineuridae, contradicting previous assumptions about a monophyletic origin of shovel-headed amphisbaenians [29].

## Conclusions

Using the pectoral region of amphisbaenians as a model system, our study provides novel insights into the relationship between muscles and bones during the reduction of shoulder girdle and limbs, indicating that muscle attachment sites that were originally connected to an ancestrally present bone shift to non-skeletal areas such as surrounding muscles, skin or connective tissue. At the same time, muscle origins themselves remain in the same region where the respective bones were ancestrally located. From this follows that pectoral muscle anatomy does not necessarily correspond to the loss or reduction of bones, indicating a decoupling of the musculoskeletal system. Also, we identified a relative anterior shift of the position of both pectoral bones and muscles relative to the ancestral position as seen in other squamates, suggesting fundamental developmental modifications during the evolution of the amphisbaenian body plan. We predict that the observed evolutionary rearrangements of shoulder muscles in worm lizards were likely driven by functional demands rather than developmental constraints. Furthermore, our study provides indirect morphological support for shovel-headed morphologies to have evolved convergently and independently across Amphisbaenia.

For future studies increased taxon sampling would be preferable, especially with respect to shovel-headed forms. In addition, studies on other limb-reduced lizard taxa, such as representatives from Scincidae or Gymnophthalmidae, may provide further, independent insights into to the modifications of the musculoskeletal system during the evolutionary transformation of the squamate body axis.

## Additional files

Additional file 1: **Table S1.** Species table. Table contains information about all specimens used in the study, abbreviations: FMNH: Florida Museum of Natural History, MfN: Museum für Naturkunde Berlin. (DOCX 14 kb)

Additional file 2: **Text S2.** Pectoral muscle morphology in Lacertidae (*Meroles cuneirostris)*. Text contains detailed information about pectoral bones and muscles in *Meroles cuneirostris*, abbreviations: cc: coracoid, cl: clavicles, dc: deltopectoral crest of humerus, hu: humerus, hy: hyoid, m b: M. brachialis, m bb: M. biceps brachii, m cbb: M. coraco-brachialis brevis, m cbl: M. coraco-brachialis longus, m dc: M. deltoideus clavicularis, m ds: M. deltoideus scapularis, m e: M. episternocleidomastoideus, m etc: M. episternocleidomastoideus and M. trapezius-complex, m ld: M. latissimus dorsi, m ls: M. levator scapulae, m o: M. omohyoideus, m p: M. pectoralis, m sas: M. serratus anterior superficialis, m sha: M. scapulo-humeralis anterior, m shp: M. scapulo-humeralis posterior, m sternc: M. sternocoracoideus, m sternh: M. sternohyoideus, m subcs: M. subcoraco-scapularis, m supc: M. supracoracoideus, m t: M. triceps, m tp: M. trapezius, ss: suprascapulae, st: sternum, v: vertebra. (DOCX 35 kb)

Additional file 3: **Figure S3.** Skull, anterior part of the vertebral column and pectoral region of *Meroles cureirostris*. A: lateral view, B: ventral view. Abbreviations: cc: coracoid, cl: clavicles, hu: humerus, hy: hyoids, icl: interclavicle, s: scapula, st: sternum, v: vertebra. (TIF 4277 kb)

Additional file 4: **Figure S4.** Pectoral muscles of *Meroles cureirostris,* A: muscles of the upper forelimb of *Meroles cureirostris*, B: superficial pectoral muscles of *Meroles cureirostris*, C: distal subjacent pectoral muscles of *Meroles cureirostris*, D: superficial and subjacent pectoral muscles of *Meroles cureirostris*, E: medial subjacent pectoral muscles of *Meroles cureirostris*. Array shows anterior direction, abbreviations: dc: deltopectoral crest of humerus, hu: humerus, m b: M. brachialis, m bb: M. biceps brachii, m cbb: M. coraco-brachialis brevis, m cbl: M. coraco-brachialis longus, m dc: M. deltoideus clavicularis, m ds: M. deltoideus scapularis, m e: M. episternocleidomastoideus, m etc: M. episternocleidomastoideus and M. trapezius-complex, m ld: M. latissimus dorsi, m ls: M. levator scapulae, m o: M. omohyoideus, m p: M. pectoralis, m sas: M. serratus anterior superficialis, m sha: M. scapulo-humeralis anterior, m shp: M. scapulo-humeralis posterior, m sternc: M. sternocoracoideus, m sternh: M. sternohyoideus, m subcs: M. subcoraco-scapularis, m supc: M. supracoracoideus, m t: M. triceps, m tp: M. trapezius. (TIF 1862 kb)

Additional file 5: **Table S5.** Pectoral muscle origins and insertions. Table contains information about pectoral muscles origins and insertions for all amphisbaenian families. (DOCX 15 kb)

## Abbreviations

(m) dc: M. deltoideus clavicularis
(m) ds: M. deltoideus scapularis
(m) etc: M. episternocleidomastoideus and M. trapezius-complex
(m) ld: M. latissimus dorsi
(m) ls: M. levator scapulae
(m) sas: M. serratus anterior superficialis
(m) shp: M. scapulo-humeralis posterior
(m) sternc: M. sternocoracoideus
(m) subcs: M. subcoraco-scapularis
(m) supc: M. supracoracoideus
cc: coracoid
cl: clavicles
dc: deltopectoral crest of humerus
FMNH: Florida Museum of Natural History
hu: humerus
hy: hyoid
IKI: iodine-potassium iodine solution
m b: M. brachialis
m bb: M. biceps brachii
m cbb: M. coraco-brachialis brevis
m cbl: M. coraco-brachialis longus
m e: M. episternocleidomastoideus
m o: M. omohyoideus
m osc: M. omohyoideus and M. sternohyoideus-complex
m p: M. pectoralis
m sha: M. scapulo-humeralis anterior
m sternh: M. sternohyoideus
m t: M. triceps
m tp: M. trapezius
MfN: Museum für Naturkunde Berlin
scc: scapulocoracoids
ss: suprascapulae
st: sternum
v: vertebra
xp: xiphoid process
