# Supplementary material for: Pectoral myology of limb-reduced worm lizards (Squamata, Amphisbaenia) suggests decoupling of the musculoskeletal system during the evolution of body elongation
Source: BMC Evol Biol. 2019 Jan 10;19:16. doi: 10.1186/s12862-018-1303-1 (PMC6329177; doi:10.1186/s12862-018-1303-1)
Supplement: Supplementary file 1 — Table S1. Species table. Table contains information about all specimens used in the study, abbreviations: FMNH: Florida Museum of Natural History, MfN: Museum für Naturkunde Berlin. (DOCX 14 kb) [file 12862_2018_1303_MOESM1_ESM.docx]

Table S1: species table

| species | collection or field number | locality | collector | date of collection | collection |
| --- | --- | --- | --- | --- | --- |
| *Meroles cuneirostris*  (Lacertidae) | field number 250/2012 | area of Gobabeb Research & Training Centre (23°34’S, 15°2’E) at the foot of dunes in *Stipagrostis sabulicola* | Sebastian Kirchhof | October 2012 | MfN |
| *Bipes biporus*  (Bipedidae) | ZMB 80325 | La Paz, Baja California, Mexico | Dr. Johannes Müller, Berlin | May 2014 | MfN |
| *Blanus strauchi*  (Blanidae) | ZMB 55008 | unknown | unknown | unknown | MfN |
| *Trogonophis wiegmanni*  (Trogonophidae) | uncatalogued | Islas Chafarinas, Spain | Dr. Jose Martin, Madrid | unknown | MfN |
| *Cynisca leucura*  (Amphisbaenidae) | ZMB 79378 | Batia (southwest barrier of Pendjari national park in Benin) | Dr. Mark-Oliver Rödel, Berlin | September 2006 | MfN |
| *Rhineura floridana*  (Rhineuridae) | UF Herp 167969 | unknown | unknown | unknown | FMNH |

Contains information about all specimens used in the study, abbreviations: FMNH: Florida Museum of Natural History, MfN: Museum für Naturkunde Berlin
