# Supplementary material for: Pectoral myology of limb-reduced worm lizards (Squamata, Amphisbaenia) suggests decoupling of the musculoskeletal system during the evolution of body elongation
Source: BMC Evol Biol. 2019 Jan 10;19:16. doi: 10.1186/s12862-018-1303-1 (PMC6329177; doi:10.1186/s12862-018-1303-1)
Supplement: Supplementary file 2 — Text S2. Pectoral muscle morphology in Lacertidae (Meroles cuneirostris). Text contains detailed information about pectoral bones and muscles in Meroles cuneirostris, abbreviations: cc: coracoid, cl: clavicles, dc: deltopectoral crest of humerus, hu: humerus, hy: hyoid, m b: M. brachialis, m bb: M. biceps brachii, m cbb: M. coraco-brachialis brevis, m cbl: M. coraco-brachialis longus, m dc: M. deltoideus clavicularis, m ds: M. deltoideus scapularis, m e: M. episternocleidomastoideus, m etc: M. episternocleidomastoideus and M. trapezius-complex, m ld: M. latissimus dorsi, m ls: M. levator scapulae, m o: M. omohyoideus, m p: M. pectoralis, m sas: M. serratus anterior superficialis, m sha: M. scapulo-humeralis anterior, m shp: M. scapulo-humeralis posterior, m sternc: M. sternocoracoideus, m sternh: M. sternohyoideus, m subcs: M. subcoraco-scapularis, m supc: M. supracoracoideus, m t: M. triceps, m tp: M. trapezius, ss: suprascapulae, st: sternum, v: vertebra. (DOCX 35 kb) [file 12862_2018_1303_MOESM2_ESM.docx]

# **Pectoral muscle morphology in Lacertidae (Meroles cuneirostris***)*

To study the pectoral muscles in *Meroles cuneirostris* we used µCT-scanning as well as manual dissections on *Meroles cuneirostris*, *Lacerta agilis* and *Lacerta spec.* combined with information from the literature. The nomenclature of the shoulder girdle muscles is equivalent to the ones used by Jenkins & Goslow [1]. Altogether, 20 muscles belong to the shoulder girdle; nine of them are associated with the superficial shoulder muscles, eight with the subjacent muscle layer, and three with the muscles of the upper forelimb.

## Bones of the shoulder girdle

The shoulder girdle apparatus of *Meroles cuneirostris* (see additional file S3) consists of cartilaginous and bony elements [2]. The shield-shaped **sternum (st)** is located on the ventral side of the specimen. It belongs to the cartilaginous parts of the shoulder and has a large sternal fontanelle from where the sternal ribs emerge. From anterior to posterior it can be separated into three topographic regions, i.e. presternum, mesosternum and xiphisternum [3]. The presternum is a large, diamond-shaped, partly calcified bony plate from where the first four sternal ribs arise. The mesosternum can be seen as the posterior elongation of the presternum and is represented by two parallel-running, mainly calcified, cartilaginous rods, which articulate with the fifth and sixth sternal ribs. The xiphisternum is located posterior to the insertion of the most posterior sternal ribs [4]. In the following description, the sternum will not be separated into pre-, meso- and xiphisternum, but treated as a single unit. The **coracoid (cc)** is located cranial to the sternum. It consists of paired cartilaginous elements, and possesses a well-developed coracoid foramen. **Scapula (s)** and **extrascapula**, both cartilaginous and in some cases also calcified elements, are located near the coracoid [5,4]. The glenoid for articulation with the humerus is formed by both coracoid and scapula. Primary bony elements of the shoulder girdle are the rod-like, delicate **clavicles (cl)**, as well as the **interclavicle (icl),** which is positioned mesial to the paired clavicles [5] and covers almost the entire middle part of the presternum in ventral view [4]. As in most lizards, the shoulder girdle starts at the level of the sixth cervical vertebra and then extends further posteriorly [6]. For reasons of completeness and because it serves as insertion side for some muscles, the **hyoid (hy)** is also marked.

In our µ-CT-scans it was hard to differentiate between cartilaginous and bony elements*.* We therefore also present µ-CT-images of another specimen of *M. cuneirostris* to better show the respective locations of the bones within the specimen. Contrary to other studies [7], staining of cartilaginous tissues using IKI-solution was not successful, and cartilaginous areas appear as dark cavities in all images. It was sometimes hard to identify tendons within the scans and dissections, so the origin and insertion areas could not always be defined at the tendon level. When there was a striking similarity to other lizard taxa, we therefore also considered information from the literature to clearly define origin and insertion sites.

## Muscles of the shoulder girdle

### Superficial muscle layers

The superficial muscles of the shoulder girdle (see additional file S4 B), which are located directly beneath the skin, are the M. latissimus dorsi, the M. deltoideus scapularis, the M. deltoideus clavicularis, the M. supracoracoideus, the M. pectoralis, the M. trapezius, the M. episternocleidomastoideus, the M. omohyoideus, and the M. sternohyoideus.

The **M. latissimus dorsi** **(m ld)** spans the dorsolateral part of the thorax as a thin layer. It connects the axial skeleton with the humerus and acts as a humeral retractor to stabilize the shoulder joint [8,9]. This dorsal, large, laminar muscle arises dorsally from an aponeurosis, which is connected to the neural spine and the ligament (inter-spine connection) of the 8^th^ cervical to the 3^rd^ lumbar vertebra [1,9]. The origin-line proceeds posteroventrally, so parallel-arranged fibres cover the entire dorsolateral ribcage. The muscle fibres of the M. latissimus dorsi run dorsoventrally and insert into the humeral joint. The M. latissimus dorsi is restricted anteriorly by the posterior edge of the M. deltoideus scapularis. Anteriorly, contrary to the information given by Gans et al. [4] for other lizards, the M. latissimus dorsi is not overlain by the M. trapezius, but both are in contact with each other in *Meroles*.

The **M. episternocleidomastoideus (m e)** is a triangular-shaped, parallel-fibred muscle that is connected to the posterior edge of the M. trapezius. Based on our findings, in *Meroles* the M. episternocleidomastoideus arises from the clavicle and extends distal to the M. levator scapulae. It runs dorsoventrally via parallel-arranged fibres where it hits the M. sternohyoideus and inserts into the coracoid. It is tapering on the way from origin to insertion. As a superficial muscle, the M. episternocleidopmastoideus is associated with the M. omohyoideus dorsally and with the M. trapezius dorsomedially.

In lateral view, the **M. trapezius** **(m tp)** is a dorsal, diffuse, superficial muscle layer with dorsoventrally oriented fibres that is positioned caudally to the M. omohyoideus and M. episternocleidomastoideus. It arises from the neural spine and runs to the anterior margin of the scapula and the dorsal half of the clavicle. The M. trapezius tapers towards its point of insertion at the acromion of the scapula and the anterodorsal part of the clavicle. It can be differentiated from other surrounding muscles by its separate point of insertion. Along its dorsal border, the M. trapezius is closely linked to the fascia of the axial musculature. Anteriorly, it can be hardly separated from the M. episternocleidomastoideus. Gans et al. [4] suggest that the anterior part of the M. latissimus dorsi is overlain by the posterior part of the M. trapezius in lizards. Such an overlay could not be found in *Meroles*. Both parts of the M. trapezius are therefore shown as a single muscle in this work.

**The M. omohyoideus** **(m o)** connects the shoulder region with the hyoid and acts as a fixator of the latter by pulling it down. It helps during breathing and swallowing and takes part in the flexion of head and neck joints [10]. The M. omohyoideus is a relatively diffuse dorsal mass that is strongly connected to the M. trapezius anteriorly and to parts of the M. episternocleidomastoideus ventrally. The orientation of the muscle fibres is cranially. The M. omohyoideus is a triangular, thin, and parallel-fibred muscle. The apex (distal tip) of the M. omohyoideus is oriented anteriorly, where it arises from the anterior part of the acromial region from the ventrolateral border of the clavicle. The M. omohyoideus proceeds towards the hyoid. Anterior to the origin of the M. omohyoideus, the M. episternocleidomastoideus is located, and posterior to the origin of the M. omohyoideus there is the M. trapezius. All three latter muscles are in contact with each other in *Meroles*. Along its anterior border, the M. omohyoideus is tightly associated with its counterpart. The M. omohyoideus represents the lateral extension of the M. episternocleidomastoideus-complex [11].

Both, M. omohyoideus and **M. sternohyoideus (m sternh)**, belong to the infrahyoidal musculature and are therefore no true pectoral muscles [12]. Their effect on the locomotory system is rather small and not very significant [4]. The M. sternohyoideus, the sternum-hyoid connector, pulls down the hyoid while swallowing. It is a slender, flat, triangular, paired, and fasciated muscle. It is located along the midline of the lower neck, and proceeds towards the hyoid [13]. It arises from the coracoid near the M. episternocleidomastoideus, to which it is also attached during its course. The insertion of the M. sternohyoideus is located medially to the insertion side of the M. omohyoideus, whereas the latter´s route is accompanied by the M. sternohyoideus in the cranial part. In the anterior, median region of the neck, both counterparts of the M. sternohyoideus are connected by soft tissue.

The **M. deltoideus scapularis (m ds)** is a humeral retractor that contributes to the general stability of the shoulder joint [8] and is also involved in humeral rotation [9]. It is a relatively large and broad, triangular-shaped muscle. During dissection the origin of the M. deltoideus scapularis at the scapula could be easily identified in *Meroles*. The M. deltoideus scapularis inserts into the humeral joint, especially into the **deltopectoral crest (dc)** of the humerus (hu, see additional file S4). Its muscle fibres run dorsoventrally and form a fan-shaped structure, which spans the lateral superficial part between scapula, suprascapula and clavicle. The M. deltoideus scapularis is located cranially to the M. latissimus dorsi. In lateral view, after removing the M. latissimus dorsi and the M. deltoideus scapularis, the M. serratus anterior superficialis is located on the dorsal edge of the scapula, caudally to the origin of M. deltoideus scapularis. The proximal M. levator scapulae is located cranially to the origin of the M. deltoideus scapularis.

The **M. deltoideus clavicularis (m dc)** is a protractor of the humerus and contributes to the general stability of the shoulder joint [8]. It shows an elongated, curved shape. Relative to the M. deltoideus scapularis, the M. deltoideus clavicularis is a rather small, ventrally-running muscle, which is located craniolaterally to the distinctly larger M. deltoideus scapularis. The point of origin is located between clavicle and the area where the clavicle and the lateral process of the interclavicle contact each other. Fibre bundles run posteriorly from the clavicle in a horizontal direction to the point of insertion on the deltopectoral crest of the humerus, where they associate anterodorsally with fibres of the M. deltoideus scapularis.

Due to its origin on the coracoid, the **M. supracoracoideus (m supc)** contributes to the supportive function of the shoulder region [14]. In terrestrial lizards it acts as a wristband muscle together with the M. deltoideus clavicularis and participates in the anterior shift of the humerus [8]. It also stabilizes the shoulder joint [1]. Its relevance for the elevation of the humerus is rather low, but it plays a significant role in humeral rotation [4]. The M. supracoracoideus, a thick, fan-shaped muscle, is closely associated with the ventrolateral part of the coracoid. According to literature, in other squamates the M. supracoracoideus has a broad origin, which expands from the anterior margin of the coracoid cartilage along the lateral surface of the coracoid cartilage to the coracoid itself, including the fibrous membrane, which spans across the coracoid fenestra [1]. Mesial fibres of the muscle run from the mesial part of the epicoracoid to the coracosternal joint. The M. supracoracoideus is closely associated with the shoulder joint. Because tendons were not visible within the scan, we further infer that the muscle inserts via a short and wide tendon into the proximal margin of the deltopectoral crest of the humerus [4]. The M. supracoracoideus lies ventromedially to the M. deltoideus clavicularis and is partly covered by the latter. The border to the M. scapulo-humeralis marks the separation line between scapula and coracoid [4]. Fibre bundles of the M. supracoracoideus run parallel to those of the M. coraco-brachialis brevis, which runs ventrally to the M. supracoracoideus and inserts distally along the deltopectoral crest of the humerus [1].

The **M. pectoralis** **(m p)** acts a retractor of the humerus [8]. It also takes part in protraction when the humerus is lifted forward and in the associated turn of the shoulder joint, but it is also involved in the fixation and turn of the scapula during locomotion, and supports breathing [14]. The M. pectoralis is a fan-shaped muscle [9] that is characterized by a superficial, thin, but relatively large muscle layer, which covers the entire chest at the level of the front limbs, and runs ventrally from the interclavicle. Fibres of the M. pectoralis arise from the interclavicle along its midline. The entire muscle is sickle-shaped and inserts with the fibres into the deltopectoral crest of the humerus. The M. pectoralis´ side of insertion is surrounded by the insertion sides of the M. deltoideus scapularis, the M. deltoideus clavicularis and the M. supracoracoideus [4].

### Subjacent muscles layers

Proximal to the superficial muscle layers the subjacent muscle layers (see additional file S4 C-E) are located. These include the M. serratus anterior superficialis, the M. scapulo-humeralis anterior, the M. scapulo-humeralis posterior, the M. levator scapulae, the M. coraco-brachialis brevis, the M. coraco-brachialis longus, the M. subcoraco-scapularis, and the M. sternocoracoideus.

The **M. serratus anterior superficialis (m sas)** separates the outer body wall from the shoulder girdle [4]. During contraction it acts as a stabilizer of the pectoral girdle [4]. The M. serratus anterior superficialis is located proximally to the M. latissimus dorsi and the M. deltoideus scapularis, by which it is also completely overlain. The muscle is located medially to the suprascapular cartilage; it arises near the deltopectoral crest of the humerus and runs to the scapula where it inserts (see additional file S4 C).

The **M. scapulo-humeralis anterior (m sha)** belongs to the protractors of the humerus [8] and consists of two parts*.* Because the M. scapulo-humeralis anterior is similar to that of other lizards [1,4], and details concerning origin and insertion sites were not visible within our µ-CT-scan, we infer from literature information that one part emerges from the anterolateral surface and the anterior margin of the scapula, the other part emerges from the lateral surface and the dorsal margin of the coracoid cartilage as well as from the coracoid above the anterior coracoid fenestra. Furthermore, corresponding to literature information both parts converge as they pass medial to the craniodorsal ligament and insert into the dorsomedial surface of the humerus proximal to the insertion of the M. latissimus dorsi. Bolk et al. [15] suggest that the M. scapulo-humeralis anterior may coalesce with the adjacent M. supracoracoideus, but this is not the case in *Meroles.* Here, both muscles run next to each other and are also partly in contact, but they do not coalesce. For reasons of simplification and better comparison, in this paper there is no further differentiation between the discrete parts of the M. scapulo-humeralis anterior, but they are treated as a single muscle.

The **M. scapulo-humeralis** **posterior (m shp)** belongs to the retractors of the humerus [8] and can act as a “single-joint-retractor” in the parasagittal plane. Jenkins & Goslow [1] showed that the M. scapulo-humeralis posterior is active during both the swing and the stand phase of the limb in *Varanus*, i.e. it supports the elevation of the humerus during the swing phase and stabilizes the shoulder joint during the stand phase [1]. The M. scapulo-humeralis posterior is much smaller than the M. scapulo-humeralis anterior. Because the M. scapulo-humeralis posterior is similar to that of other lizards [8,1,4], and because the muscle origins were not visible in detail in the µ-CT-scan, we infer from literature information that it arises from the posterior half of the lateral surface of the scapula and suprascapula and from the posterior margin of the scapula, covering two thirds of the lateral surface of the bone. The M. scapulo-humeralis posterior lies deep to the M. deltoideus scapularis, dorsally to the scapular origin of the M. triceps. Fibres run ventrally and lie deep to the scapular part of the M. triceps. Because tendons were not visible within the scan, we further infer that the muscle inserts via a robust tendon into the dorsal surface of the small tubercle of the humerus, distal to the insertion side of the M. subcoraco-scapularis [1,4]. In some lizards, e.g. *Iguana,* there is no M. scapulo-humeralis posterior, or it has been mistakenly identified as the medial head of M. subcoraco-scapularis [4].

Together with other parts of the dorsal axial somatic complexes, the **M. levator scapulae (m ls)** supports lateral undulation during locomotion. It spans the acromioclavicular joint and helps to stabilize the entire pectoral girdle complex as a unit. It also stabilizes the scapulocoracoid [4]. Functionally, the M. levator scapulae helps to pull the scapula anteriorly [14] during locomotion and forms a functional unit with the surrounding muscles, i.e. the M. serratus anterior and the M. serratus anterior superficialis. In *Meroles*, our µ-CT scans imply that the M. levator scapulae seems to arise from a ventral area of the cranium. The M. levator scapulae is a bulky muscle [9] that, according to literature, consists of two separate parts, a dorsal and a ventral one. Because the M. levator scapulae of *Meroles* is similar to that of other lizards [1,4] and because the origin of the latter muscle was not visible in detail in our µ-CT scans, we infer that the larger dorsal part arises from the lateral process of the atlas and from an aponeurosis, which covers the epaxial musculature and spreads from the atlas to the 4^th^ cervical [1]. It fans out posteriorly and inserts into the entire lateral surface of the anterior margin of the suprascapula [1,4]. The ventral part of M. levator scapulae runs laterally and ventrally. We further infer that it arises from the same lateral process of the atlas as the dorsal part, and some of its fibers insert into the anterior margin of the scapula, whereas the remaining fibres insert into the dorsal margin of the scapula [1]. The M. levator scapulae lies proximally to the M. omohyoideus, the M. sternohyoideus and the M. episternocleidomastoideus, by which it is also largely covered in lateral view. For reasons of simplification, there is no differentiation in this paper between the two parts of the M. levator scapulae.

The **M. coraco-brachialis brevis (m cbb)** is an adductor and a posterior rotator of the humerus during retraction [8]. It also plays an important role in the stabilization of the shoulder joint [8,4]. The M. coraco-brachialis brevis is the anterior part of the M. coracobrachialis-complex [4]. Because this large muscle is similar to that of other lizards, and because its origin was not visible in detail in our µ-CT scans we infer that it arises from the posterior two thirds of the ventral surface of the coracoid as well as from the tissue above the posterior coracoid fenestra and from the posterior margin of the coracoid, from where it spans an area ventrally to the shoulder joint. Some fibres also emerge from the coracoid cartilage [1]. It spans the coracosternal and the glenohumeral joint (shoulder joint) and expands posteriorly to the most posterior part of the coracoid. In surface view, the M. coraco-brachialis brevis has a triangular shape [4]. The posterior part of the muscle´s origin lies deep to that of the M. biceps. Fibre bundles run parallel to each other and insert into the deltopectoral crest of the humerus via a flat tendon, as well as into the proximal half of the humeral shaft along the anteroventral surface [1]. Fibres also run dorsally and ventrally and insert into the ventral part of the humerus. The insertion side is positioned within a groove between the insertion side of the M. pectoralis on the deltopectoral crest and a line extending distally from the medial part of the humeral articular surface [4].

The **M. coraco-brachialis longus (m cbl)** is a strong humeral retractor [16,8]. This muscle acts during the first phase of retraction, when the limb is pulled. The M. coraco-brachialis longus is the posterior part of the M. coracobrachialis-complex. It lies deep to the M. coraco-brachialis brevis [4]. The origin of the M. coraco-brachialis longus is posterior to that of the M. coraco-brachialis brevis. Because the M. coraco-brachialis longus is similar to that of other lizards, we infer that the superior part of the muscle arises from the medial part of the posterior half of the coracoid, whereas the inferior part emerges from the posterior end of the coracoid. Fibres of the superior part insert via a flat tendon that runs to the entepicondyle. Further, we infer that the inferior part is linked to this tendon as well as directly to the entepicondyle [1]. During its course it makes a turn and fans out distally. The insertion side is located between the insertion sides of the M. brachialis and the M. triceps [4]. According to its fibre architecture, the M. coraco-brachialis longus is more complex than the M. coraco-brachialis brevis. Fibres either show a fleshy or tendinous origin, while most fleshy origins are attached to the coracoid process [4]. In this paper there is no differentiation between the two parts of the muscle.

The **M. subcoraco-scapularis (m subcs)** is a single-joint retractor and a rotator of the humerus that helps in stabilizing the shoulder joint [8,4]. It is an aggregation of the M. subcoracoideus and the M. subscapularis [9]. The **M. subscapularis** supports retraction and rotation of the humerus, whereas the **M. subcoracoideus** stabilizes the shoulder girdle during humeral protraction. The M. subcoraco-scapularis is positioned proximal to the M. coraco-brachialis longus and M. coraco-brachialis brevis. It is surrounded by the M. scapulo-humeralis posterior laterally and by the M. sternocoracoideus pectorally. It arises near the coracoid and runs to the humerus where it inserts.

The **M. sternocoracoideus (m sternc)** functionally supports the sternum and coracoid [17]. Peterson [8] states that the muscle acts across the coracosternal joint. The M. sternocoracoideus supports holding the relative position of the coracoid and the sternum during humeral retraction [4]. This muscle is positioned within the pectoral region proximally to the M. coraco-brachialis longus and the M. pectoralis. It arises from the sternum and runs to the coracoid.

### Muscles of the upper forelimb

The muscles of the upper forelimb (see additional file S4 A) are the M. triceps, the M. brachialis and the M. biceps brachii.

During locomotion the **M. biceps brachii** **(m bb)** is active during the stand phase of the humerus, i.e. when the limb is fixed to the ground and while the body is still moving. It flexes the elbow and is responsible for the stabilization of the shoulder joint [1]. The M. biceps brachii is similar to that of other lizards. It arises, consisting of two parts, from the lateral surface of the coracoid along a narrow area next to the coracosternal joint [18,19]. The anterior part is fleshy and runs proximally to the anterior part of M. pectoralis. Its fibres converge into an inferred narrow tendon, which is connected to the distal part of the M. biceps. The posterior part consists of an inferred broad tendon, which runs proximally to the M. pectoralis. The long, distal portion of the posterior part has two insertion sides: it expands to the radial side of the forearm flexors and inserts via an inferred tendon that is shared with the M. brachialis into the proximal part of the ulna [1]. All tendon-linked insertions have to be described as inferred, because tendons were not visible within the µ-CT scan. In the present paper, the division of the M. biceps brachii is not taken into consideration, and both parts are treated as a single muscle.

The **M. brachialis (m b)** is responsible for the flexion and tension of the elbow joint [4]. Its antagonist is the M. triceps [12]. During locomotion, the M. brachialis is active during the swing phase of the humerus when the limb is unfastened to the ground and moved anteriorly [1]. The M. brachialis is the smallest and thinnest of the three muscles of the arm. It exhibits a lot of complex insertion sides and is similar to that of other lizards. It arises from the lateral (anterior) surface of the humeral shaft and extends distally from the deltopectoral crest of the humerus up to two thirds of the humeral proximodistal length. Another insertion side is on an aponeurosis, which is located between the M. brachialis and the lateral part of the M. triceps. The M. brachialis arises from a point delimited by the coalescence of the insertion sides of the M. deltoideus scapularis, the M. deltoideus clavicularis, the M. supracoracoideus, the M. pectoralis and the dorsal border of the M. biceps brachii, as well as the ventral border of the lateral humeral head of the M. triceps [4]. Because tendons were not visible within our µ-CT scan, we infer that most muscle fibres of the M. brachialis are connected to a tendon of the M. biceps, which runs proximally to the ulna. We also infer that some fibres converge into a narrow tendon inserting into the proximoposterior surface of the radius [1]. Distally, the M. brachialis and the M. biceps brachii converge and fibres of both muscles run into a broad aponeurosis.

The **M. triceps (m t)** is responsible for the extension of the elbow [8]; its antagonist is the M. brachialis [12]. The M. triceps is a long, parallel-fibred muscle that covers the entire humerus. It arises from the deltopectoral crest of the humerus and runs along the humerus to insert via an intramuscular tendon [5] into the olecranon.

# List of abbreviations

cc: coracoid

cl: clavicles

dc: deltopectoral crest of humerus

hu: humerus

hy: hyoid

icl: interclavicle

m b: M. brachialis

m bb: M. biceps brachii

m cbb: M. coraco-brachialis brevis

m cbl: M. coraco-brachialis longus

m dc: M. deltoideus clavicularis

m ds: M. deltoideus scapularis

m e: M. episternocleidomastoideus

m etc: M. episternocleidomastoideus and M. trapezius-complex

m ld: M. latissimus dorsi

m ls: M. levator scapulae

m o: M. omohyoideus

m osc: M. omohyoideus and M. sternohyoideus- complex

m p: M. pectoralis

m sas: M. serratus anterior superficialis

m sha: M. scapulo-humeralis anterior

m shp: M. scapulo-humeralis posterior

m sternc: M. sternocoracoideus

m sternh: M. sternohyoideus

m subcs: M. subcoraco-scapularis

m supc: M. supracoracoideus

m t: M. triceps

m tp: M. trapezius

s: scapula

st: sternum

v: vertebra

## References

1. Jenkins FA, Goslow GE. The functional anatomy of the shoulder of the savannah monitor lizard (*Varanus exanthematicus*). Journal of Morphology. 1983;175(2):195-216.
2. Fürbringer M. Zur Vergleichenden Anatomie des Brustschulterapparates und der Schultermuskeln IV. Jena Z Naturwiss. 1900;34:215-718.
3. Parker WK. A monograph on the structure and development of the shoulder-girdle and sternum in the Vertebrata. Vol. 42. London: Ray Society; 1868.
4. Gans C, Gaunt AS, Adler K. Biology of the Reptilia. The Skull and Appendicular Locomotor Apparatus of Lepidosauria. Vol. 21, Morphology I. Ithaca, New York, USA: Society for the Study of Amphibians and Reptiles; 2008.
5. Westheide W, Rieger R. Spezielle Zoologie. Teil 2: Schädel- oder Wirbeltiere. 2nd ed. Heidelberg: Spektrum Akademischer Verlag; 2010.
6. Kearney M. Appendicular Skeleton in Amphisbaenians (Reptilia: Squamata). Copeia. 2002;3:719-38.
7. Metscher BD. MicroCT for developmental biology: A versatile tool for high-contrast 3D imaging at histological resolutions. Developmental Dynamics 2009;238**(**3):632–640.
8. Peterson JA. Adaptation for arboreal locomotion in the shoulder region of lizards. University of Chicago Press. 1973.
9. Abdala V, Diogo R. Comparative anatomy, homologies and evolution of the pectoral and forelimb musculature of tetrapods with special attention to extant limbed amphibians and reptiles. J Anat. 2010;217:236-573.
10. Pabst R, Putz R. Sobotta, Atlas der Anatomie des Menschen. München, Jena: Urban und Fischer in Elsevier; 2000.
11. Avery DF, Tanner WW. Evolution of the iguanine lizards (Sauria, Iguanidae) as determined by osteological and myological characters. Brigham Young University Science Bulletin. Biological Series. 1971;12(3):1.
12. Salomon FV, Geyer H, Gille U. Anatomie für die Tiermedizin. 2nd ed. Stuttgart: Enke Verlag; 2008.
13. Rüdinger N. Die Muskeln der vorderen Extremitäten der Reptilien und Vögel. Verh. d. Hollandsche Maatschappi van Wetenschappen to Haarlem; 1868.
14. Starck D. Vergleichende Anatomie der Wirbeltiere auf evolutionsbiologischer Grundlange. Das Skeletsystem. Allgemeines, Skeletsubstanzen, Skelet der Wirbeltiere einschließlich Lokomotionssystem. 2nd ed. Berlin, Heidelberg, New-York: Springer-Verlag; 1979.
15. Bolk L, Göppert E, Kallius E, Lubosch W. Handbuch der vergleichenden Anatomie der Wirbeltiere*.* 5th ed. Berlin: Urban und Schwarzenberg; 1938.
16. Vialleton L. Membres et Ceintures des Vertébrés Tétrapodes. Critique Morphologique de Transformisme, Paris: Librairie Octave Doin; 1924.
17. George JC. The muscular system of *Uromastix hardwickii* Gray. Bombay Univ. J. 1948;17:1-23.
18. Holmes R. The osteology and musculature of the pectoral limb of small captorhinids. J Morphol. 1977;152:101-40.
19. Dilkes DW. Appendicular myology of the hadrosaurian dinosaur *Maiasaura peeblesorum* from the Late Cretaceous (Campanian) of Montana. Transactions of the Royal Society of Edinburgh Earth Sciences. 2000;90:87-125.
