# Supplementary figures and images for: Pectoral myology of limb-reduced worm lizards (Squamata, Amphisbaenia) suggests decoupling of the musculoskeletal system during the evolution of body elongation
Source: BMC Evol Biol. 2019 Jan 10;19:16. doi: 10.1186/s12862-018-1303-1 (PMC6329177; doi:10.1186/s12862-018-1303-1)

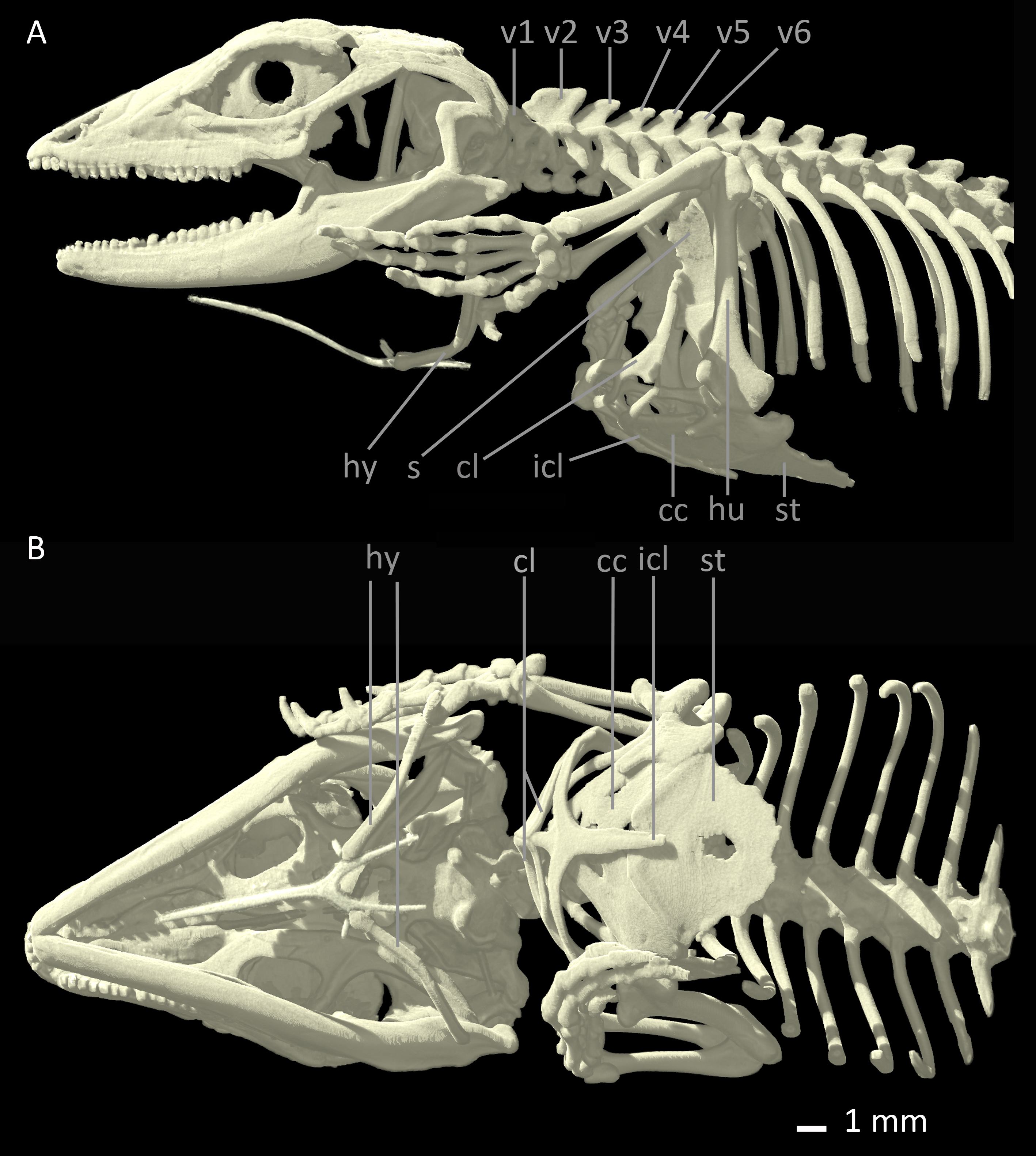

Supplement: Supplementary file 3 — Figure S3. Skull, anterior part of the vertebral column and pectoral region of Meroles cureirostris. A: lateral view, B: ventral view. Abbreviations: cc: coracoid, cl: clavicles, hu: humerus, hy: hyoids, icl: interclavicle, s: scapula, st: sternum, v: vertebra. (TIF 4277 kb) [file 12862_2018_1303_MOESM3_ESM.tif]

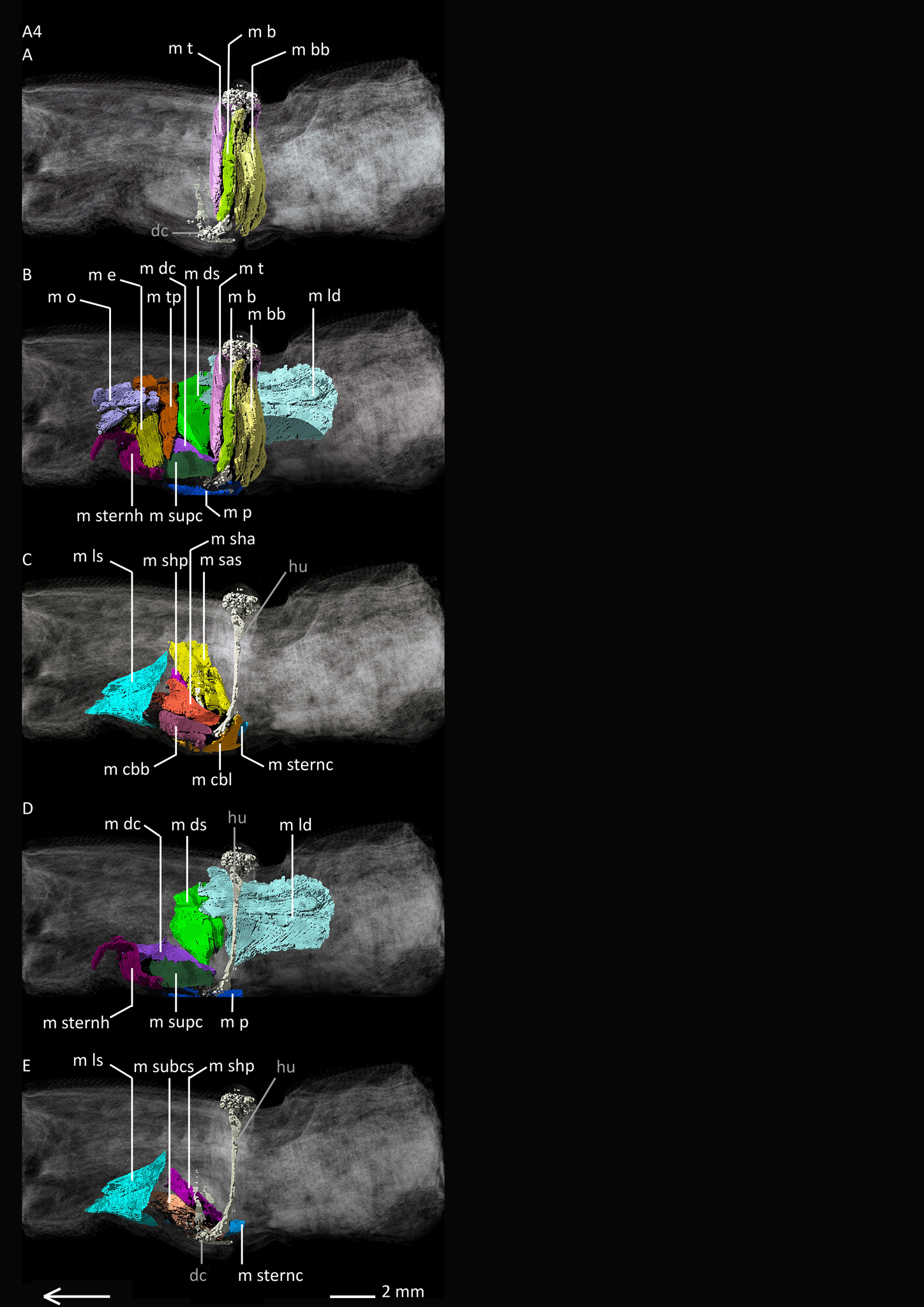

Supplement: Supplementary file 4 — Figure S4. Pectoral muscles of Meroles cureirostris, A: muscles of the upper forelimb of Meroles cureirostris, B: superficial pectoral muscles of Meroles cureirostris, C: distal subjacent pectoral muscles of Meroles cureirostris, D: superficial and subjacent pectoral muscles of Meroles cureirostris, E: medial subjacent pectoral muscles of Meroles cureirostris. Array shows anterior direction, abbreviations: dc: deltopectoral crest of humerus, hu: humerus, m b: M. brachialis, m bb: M. biceps brachii, m cbb: M. coraco-brachialis brevis, m cbl: M. coraco-brachialis longus, m dc: M. deltoideus clavicularis, m ds: M. deltoideus scapularis, m e: M. episternocleidomastoideus, m etc: M. episternocleidomastoideus and M. trapezius-complex, m ld: M. latissimus dorsi, m ls: M. levator scapulae, m o: M. omohyoideus, m p: M. pectoralis, m sas: M. serratus anterior superficialis, m sha: M. scapulo-humeralis anterior, m shp: M. scapulo-humeralis posterior, m sternc: M. sternocoracoideus, m sternh: M. sternohyoideus, m subcs: M. subcoraco-scapularis, m supc: M. supracoracoideus, m t: M. triceps, m tp: M. trapezius. (TIF 1862 kb) [file 12862_2018_1303_MOESM4_ESM.tif]
