# Supplementary material for: Pectoral myology of limb-reduced worm lizards (Squamata, Amphisbaenia) suggests decoupling of the musculoskeletal system during the evolution of body elongation
Source: BMC Evol Biol. 2019 Jan 10;19:16. doi: 10.1186/s12862-018-1303-1 (PMC6329177; doi:10.1186/s12862-018-1303-1)
Supplement: Supplementary file 5 — Table S5. Pectoral muscle origins and insertions. Table contains information about pectoral muscles origins and insertions for all amphisbaenian families. (DOCX 15 kb) [file 12862_2018_1303_MOESM5_ESM.docx]

| **Superficial muscles** |  | ***Bipes biporus*** | ***Blanus strauchi*** | ***Trogonophis wiegmanni*** | ***Cynisca leucura*** |
| --- | --- | --- | --- | --- | --- |
| M. latissimus dorsi | origin | dorsal layer of connective tissue as well as from the surface of the M. episternocleidomastoideus and M. trapezius-complex | dorsal layer of connective tissue near the skin | dorsal layer of connective tissue near the skin | dorsal layer of connective tissue near the skin |
|  | insertion | deltopectoral crest of the humerus | ventral layer of connective tissue near the skin | ventral layer of connective tissue near the skin | ventral layer of connective tissue near the skin and surface of the M. deltoideus clavicularis |
| M. episternocleidomastoideus and trapezius complex | origin | dorsal layer of connective tissue near the skin | connective tissue situated between the M. deltoideus scapularis and the outer body wall | vertebral column | near the vertebral column |
|  | insertion | near the cranium | near the cranium | near the cranium | near the cranium |
| M. omohyoideus and sternohyoideus complex | origin | upper third of the lateral body half from an inter-muscular area of connective tissue | M. omohyoideus: dorsal region from a layer of connective tissue situated between the M. deltoideus scapularis and the M. episternocleidomastoideus and M. trapezius-complex  M. sternohyoideus:  layer of connective tissue near the M. deltoideus scapularis | scapulocoracoid | surface of the M. coraco-brachialis longus and into M. supracoracoideus |
|  | insertion | hyoid | M. omohyoideus:  into a layer of connective tissue near the M. pectoralis, into the hyoid and also near the cranium  M. sternohyoideus:  cranium at the level of the hyoid | hyoid | into the hyoid and into an area further dorsally, near the cranium |
| M. deltoideus scapularis | origin | suprascapula | dorsally from a sheet of connective tissue beneath the skin | surface of the M. levator scapulae and M. longissimus | surface of the M. levator scapulae as well as from a large part of the surface of the M. longissimus |
|  | insertion | deltopectoral crest of the humerus | muscle strand near the cranium at the level of the braincase near the inner ear | area of connective tissue near the cranium | area of connective tissue near the cranium |
| M. deltoideus clavicularis | origin | suprascapula | undifferentiated muscle near the first rib | scapulocoracoid | surface of the M. deltoideus scapularis |
|  | insertion | scapulocoracoid, surface of the M. supracoracoideus anteriorly and M. deltoideus scapularis posteriorly | layer of connective tissue between the M. serratus anterior superficialis and the M. deltoideus scapularis | area near the cranium | into the surface of the M. supracoracoideus |
| M. supracoracoideus | origin | scapulocoracoid | near the ribs | scapulocoracoid | fibrously from the skin, i.e. connective tissue beneath the skin in the lateral area of the body, at the level of the scapulocoracoids |
|  | insertion | deltopectoral crest of the humerus | cranium | near the cranium | near the cranium |
| M. pectoralis | origin | layer of connective tissue between skin and sternum | hyoid | scapulocoracoid near the sternum | ventral layer of connective tissue near skin |
|  | insertion | deltopectoral crest of the humerus | connective tissue near the skin | layer of connective tissue near the skin | dorsal layer of connective tissue and surface of the M. episternocleidomastoideus and M. trapezius complex (anterior) surface of M. latissimus dorsi (posterior) |
| **Subjacent muscles** |  |  |  |  |  |
| M. serratus anterior superficialis | origin | suprascapula | surface of the M. deltoideus scapularis | posterior to the origin of the M. levator scapulae on the first vertebra | posteroproximally from the origin of the M. levator scapulae from the first vertebra |
|  | insertion | deltopectoral crest of the humerus | inserts into a layer of connective tissue near the M. levator scapulae and the vertebral column | near the scapulocoracoid on the surface of the M. scapulo-humeralis anterior and the M. scapulo-humeralis posterior | layer of inter-muscular connective tissue |
| M. scapulo-humeralis anterior | origin | clavicle and scapulocoracoid | at the level of the first ribs | scapulocoracoid | scapulocoracoid |
|  | insertion | deltopectoral crest of the humerus | into a layer of connective tissue between the M. serratus anterior superficialis and the vertebral column | surface of M. serratus anterior superficialis | surface of M. scapulo-humeralis posterior |
| M. scapulo-humeralis posterior | origin | scapulocoracoid | scapulocoracoid | near the scapulocoracoid | scapulocoracoid |
|  | insertion | surface of the adjacent muscles, i. e. M. subcoraco-scapularis, M. coraco-brachialis brevis and M. coraco-brachialis longus | vertebral column near the end of the second vertebra | insert into an area of connective tissue near the M. coraco-brachialis brevis | surface of M. serratus anterior superficialis |
| M. levator scapulae | origin | lateral process of the atlas | cranial origin near the hyoid | inter-muscular area near the lower level of the cranium | cranium |
|  |  | suprascapula | surface of M. supracoracoideus | surface of M. longissimus | surface of M. longissimus |
| M. coraco-brachialis brevis | origin | Inter-muscular connective tissue | between the first and second rib | connective tissue | scapulocoracoid |
|  | insertion | connective tissue and surface of M. coraco-brachialis longus | connective tissue next to the vertebral column | connective tissue | into the M. sternocoracoideus |
| M. coraco-brachialis longus | origin | scapulocoracoid | surface of M. supracoracoideus | ventral region of the body near the skin | surfaces of M. supracoracoideus and M. coraco-brachialis brevis |
|  | insertion | deltopectoral crest of the humerus | vertebral column near the M. subcoraco-scapularis | layer of connective tissue near the origin of the M. levator scapulae | surface of the M. omohyoideus and sternohyoideus-complex, and M. supracoracoideus |
| M. subcoraco-scapularis | origin | ventral area, layer of connective tissue inside the body | vertebral column | larger part: area of connective tissue near the M. deltoideus clavicularis  smaller part: surface of M. omohyoideus and M. sternohyoideus-complex | ventral area, i.e. from a layer of connective tissue inside the body |
|  | insertion | deltopectoral crest of the humerus | vertebral column | larger part: intramuscular bundle of connective tissue inside the body near M. serratus anterior superficialis  smaller part: inside connective tissue situated between the M. scapulo-humeralis posterior and the M. serratus anterior superficialis | scapulocoracoid and surface of the M. levator scapulae |
| M. sternocoracoideus | origin | layer of connective tissue near the sternum | surface of the M. coraco-brachialis brevis | sternum | layer of connective tissue near M. omohyoideus and M. sternohyoideus complex |
|  | insertion | deltopectoral crest of the humerus | surface of M. scapulo-humeralis anterior and M. coraco-brachialis brevis | scapulocoracoid | surface of M. coraco-brachialis brevis |
